# RNA binding protein HuD promotes autophagy and tumor stress survival by suppressing mTORC1 activity and augmenting ARL6IP1 levels

**DOI:** 10.1186/s13046-021-02203-2

**Published:** 2022-01-10

**Authors:** Kausik Bishayee, Khadija Habib, Uddin Md. Nazim, Jieun Kang, Aniko Szabo, Sung-Oh Huh, Ali Sadra

**Affiliations:** 1 Department of Pharmacology, College of Medicine, Institute of Natural Medicine, Hallym University, Chuncheon, South Korea; 2 Department of Anatomy, Alfaisal University, College of Medicine, Riyadh, Kingdom of Saudi Arabia

**Keywords:** ELAVL4, GRB-10, ARL6IP1, mTORC1, Cancer cell survival

## Abstract

**Background:**

Neuronal-origin HuD (ELAVL4) is an RNA binding protein overexpressed in neuroblastoma (NB) and certain other cancers. The RNA targets of this RNA binding protein in neuroblastoma cells and their role in promoting cancer survival have been unexplored. In the study of modulators of mTORC1 activity under the conditions of optimal cell growth and starvation, the role of HuD and its two substrates were studied.

**Methods:**

RNA immunoprecipitation/sequencing (RIP-SEQ) coupled with quantitative real-time PCR were used to identify substrates of HuD in NB cells. Validation of the two RNA targets of HuD was via reverse capture of HuD by synthetic RNA oligoes from cell lysates and binding of RNA to recombinant forms of HuD in the cell and outside of the cell. Further analysis was via RNA transcriptome analysis of HuD silencing in the test cells.

**Results:**

In response to stress, HuD was found to dampen mTORC1 activity and allow the cell to upregulate its autophagy levels by suppressing mTORC1 activity. Among mRNA substrates regulated cell-wide by HuD, GRB-10 and ARL6IP1 were found to carry out critical functions for survival of the cells under stress. GRB-10 was involved in blocking mTORC1 activity by disrupting Raptor-mTOR kinase interaction. Reduced mTORC1 activity allowed lifting of autophagy levels in the cells required for increased survival. In addition, ARL6IP1, an apoptotic regulator in the ER membrane, was found to promote cell survival by negative regulation of apoptosis. As a therapeutic target, knockdown of HuD in two xenograft models of NB led to a block in tumor growth, confirming its importance for viability of the tumor cells. Cell-wide RNA messages of these two HuD substrates and HuD and mTORC1 marker of activity significantly correlated in NB patient populations and in mouse xenografts.

**Conclusions:**

HuD is seen as a novel means of promoting stress survival in this cancer type by downregulating mTORC1 activity and negatively regulating apoptosis.

**Supplementary Information:**

The online version contains supplementary material available at 10.1186/s13046-021-02203-2.

## Background

Neuroblastoma (NB) is a tumor that originates from the peripheral sympathetic nerve tissue and it is the most prevalent solid tumor of childhood, being metastasized by the time it is diagnosed [1]. The neuron-specific RNA binding protein Hu Antigen D (HuD) (also known as Embryonic Lethal, Abnormal Vision, Drosophila Like RNA Binding Protein 4 (ELAVL4)) is overexpressed in a number of cancer types, notably NB and small cell lung cancer [2–4]. As an RNA binding protein, HuD has numerous roles in affecting RNA processing, translation and stability. HuD is also involved in transport of various RNAs [5]. The role of HuD in developing neurons, adult neurons and a few other non-cancer types including pancreatic β cells has been fully documented; however, its role in neuroblastoma remains to be defined [6–8].

HuD is part of a family of RNA binding proteins, namely HuB, HuC, and HuR [2, 9], containing three RNA-binding domains (RBDs) and a conserved linker region, separating the last RBD from the rest of the molecule. For interactions with various proteins, HuD and family members can form homo- and heteromultimers with each other [10]. HuD also promotes cap-dependent translation of mRNA, by binding to Eukaryotic Translation Initiation Factor 4A1 (eIF4A) protein that is yet poly(A)-dependent [11]. HuD physically interacts with the light chain of microtubule-associated protein Microtubule Associated Protein 1B (MAP1B) [12] and it interacts with TAP/NXF1, the primary export receptor for the bulk mRNA [13]. HuD has also been reported to interact with small non-coding RNA Y3 as part of regulating its interaction with HuD RNA targets [14]. In neurons, HuD levels are under the influence of micro-RNA miR375 as part of regulation of their differentiation [15] with miR375 levels under the control of Neurogenic Differentiation Factor 1 (NeuroD1; ND1) [16–18]. The above findings in regards to the RNA substrates for HuD and their significance have only focused on neurons and other non-transformed cells.

As the role of overexpressed HuD in NB is not known, and being an RNA binding protein, we sought to define the mRNA targets for HuD in these cells and whether they have a role in cellular survival. HuD turned out to become heavily upregulated when the cells are put under various forms of stress (mimicked hypoxia, serum starvation and ER stress). One of the hallmarks of stress survival is the shifting of the cell’s anabolic processes to catabolic ones with down modulation of the Mechanistic Target of Rapamycin mTOR Complex 1 (mTORC1) activity [19]. In our hands, the protein and mRNA level changes for HuD coincided with inverse changes for mTORC1 activity under starvation or growth. A targeted knockdown of HuD reduced cell viability and survival under stress. From RNA profiling and function assays, we found stress survival due to upregulated HuD occurring via two of its substrates, Growth Factor Receptor Bound Protein 10 (GRB-10) and ADP Ribosylation Factor Like GTPase 6 Interacting Protein 1 (ARL6IP1). Via GRB-10, HuD downregulates mTORC1 activity and increases markers of autophagy by disrupting Raptor-mTOR kinase interaction. HuD also promotes survival by binding and stabilizing ARL6IP1 RNA message, an ER-resident apoptosis-resistance factor. For GRB-10 and ARL6IP1, their cell-wide mRNA levels significantly correlate with HuD mRNA levels in NB patient populations and mouse xenografts. Additionally, we uncovered counterbalance regulation of HuD levels from the mTOR environmental sensory network as mTORC1 levels increase NeuroD1 (ND1) transcription factor activity, which in turn increases miR375 levels, a negative modulator of HuD. From this work, HuD is a novel negative modulator of mTORC1, lifting mTOR suppression of autophagy and promoting survival. As a therapeutic target, HuD is demonstrated to be required for tumor growth in NB xenograft models with its loss leading to growth inhibition.

## Methods

### Cancer patient and cell line dataset analysis

Expression of HuD, GRB-10, ARL6IP1, ACTB, and GAPDH across different cancer types were curated from datasets available in R2 (R2: microarray analysis and visualization platform; http://r2.amc.nl; Department of Oncogenomics, Academic Medical Center). The data was plotted as mean ± SD. The detail of the datasets is listed in Table S1. Data for HuD expression in different cancer cell lines were obtained from the CCLE dataset (https://portals.broadinstitute.org/ccle).

### Cell culture

Cells were grown at 37 °C in a humidified 5% CO_2_ incubator. They were cultured in their respective media supplemented with 10% fetal bovine serum (FBS). The cell lines used were periodically checked for mycoplasma infection using Universal Mycoplasma Detection Kit (ATCC 30-1012 K) (ATCC) and we found no cells that were infected. The cell lines are listed in Table S2.

### Transfection of shRNA, siRNA, microRNAs, overexpression, and control vectors

Transfection was performed according to the manufacturer’s protocol. Briefly, the cells were seeded in 6-well or 96-well culture plates in growth medium without antibiotics. Lipofectamine-3000 and P3000 reagent (Thermo Fisher) were used for transfection. The plasmids used in the study are listed in Table S3. Accession numbers for the constructs were as follows: HuD (Elavl4) NM_001144777, GRB-10 (Grb10) NM_001303422, and ARL6IP1 (Arl6ip1) NM_015161.

### Inducible HuD shRNA expressing stable cell line preparation and lentivirus transduction

The SMARTvector inducible HuD shRNA Lenti-particles were purchased from Horizon-Dharmacon (CO, USA) to prepare stable cell lines; the cells were transduced with doxy-inducible HuD shRNA lentivirus particles or HuD or control shRNA lentivirus particles (MOI 2.0) in presence of polybrene (5 μg/ml). After incubation for 48 h, cells were assayed for viability changes. For preparing stable cell lines, transduced cells were selected with puromycin after 72 h post-transduction and continued for 7 days. Doxycycline was used for inducing shRNA. The details of the lentiviral particles used are listed in Table S4.

### Mouse cortical neuron isolation and culture

Six-week-old mice were purchased from DBL Korea (South Korea). The mice were sacrificed and their cortical neurons were isolated following a conventional protocol with cell pellets resuspended in Neurobasal A media (Invitrogen 21103049), supplemented with B27 (2%) and penicillin/streptomycin. Experiments involving animals were approved by the Institutional Animal Care and Use Committee (IACUC) of Hallym University, Chuncheon, South Korea (approval number, Hallym2020–19).

### Viability assays

Cells were plated in a 96-well plate at a density of 3 × 10^4^ cells/well and after treatment, the assay was performed using ATCC ReliaBlue Cell Viability Reagent (30–1014, ATCC). Fluorescence (RFUs/absorbance at 570 nm) was measured and viability histograms were plotted using GraphPad Prism 5 software (GraphPad). All the experiments were conducted 2 or more times for reproducibility.

### RNA extraction and qPCR / sequencing

Total mRNA was extracted using the miRNeasy Mini Kit (Qiagen), from which cDNA was made using the miScript II RT Kit (Qiagen) and according to the manufacturer’s protocol. Levels of target message RNA were detected and quantified with SYBR Green Reverse Transcriptase Polymerase Chain Reaction (RT-PCR) kit (Qiagen). A list of PCR primers are listed in Table S5. For sequencing, extracted RNA was sent to eBiogen Microarray Service (eBiogen, Seoul, South Korea) using Affymetrix Human Gene 2.0 ST Array profiling (Affymetrix, Thermo Fisher). Expression data were calculated as fold changes relative to control.

### RNA immunoprecipitation assay and sequencing (RIP-SEQ)

The RNA immunoprecipitation assays and sequencing were performed according to the conventional protocol, listed in detail in the supplementary methods section. Extracted RNAs were then either sent for sequencing to eBiogen (eBiogen, Seoul, South Korea), or cDNA was prepared using the miScript II RT Kit (Qiagen) and qPCR was performed as above.

### Immunoprecipitation

Cells were lysed on ice for 30 min in Nonidet P40 (NP40) buffer (50 mM Tris-HCl, pH 7.4, 250 mM NaCl, 5 mM NaF, 1 mM Na_3_VO_4_, 1% NP40, 0.02% NaN_3_) containing a protease inhibitor cocktail (Roche). After the pre-clearing step, lysates were then further incubated with anti-primary antibody (anti-GRB-10, Raptor, DYKDDDDK (FLAG)) overnight at 4 °C with 20 μl protein A-agarose in 2 μg of antibodies. Samples were eluted from the beads by the addition of 50 μl 1X SDS sample buffer, immediately boiled, and were separated on SDS-PAGE gels for Western blot analysis.

### Protein extraction and Western blotting

Cells were seeded in 6-well plates at 1 × 10^6^ cells per well. The treated cells were lysed on ice in radioimmunoprecipitation assay (RIPA) lysis buffer (150 mM NaCl, 1% NP-40, 0.5% sodium deoxycholate, 0.1% SDS, 50 mM Tris-HCl, pH 8) that included a cocktail of protease inhibitors (Roche). A conventional Western blot experiment was performed and bands were visualized by enhanced chemiluminescence (ECL) (Luminata Forte) (Millipore) either by film or by detection with FUSION FX-Western Blot & Chemi imaging system (Vilber Lourmat). Antibodies used for Western blotting and IP are listed in Table S6.

### RNA half-life assays

IMR-32 shHuD inducible cells were plated and shHuD was induced by doxycycline (2 μg/ml) treatment or HEK293 cells transfected with control plasmid or shHuD and/or FLAG-HuD OX plasmids for 24 h. The cells were incubated and treated with freshly prepared actinomycin D (5 μg/ml) added to the culture to stop the transcription process. Total mRNA levels from the cells were determined by qPCR and normalized by GAPDH; qPCR for HDAC2 reads was used as a negative control.

### GST HuD pull-down assays for RNA stability

IMR-32 cells were lysed in polysome lysis buffer and lysates were then precleared with 100 μl of glutathione−agarose (GA) beads (Millipore G4510) preloaded with 10 μg glutathione S-transferase (GST) for 2 h at 4 °C. Next, an RNA precipitation was performed with 20 μl of GA beads preloaded with 2 μg GST (Entrez Gene ID: 2944; NBC1–18537, NovusBio) or 4 μg GST-HuD (giving equivalent molar amount as the control GST) (Entrez Gene ID: 1996; H00001996-Q01, NovusBio). Expression of HuD binders-GRB-10 and ARL6IP1 and nonbinder control, HDAC2, was performed in control and test samples by qPCR Systems. All the experiments were conducted 2 or more times for reproducibility.

### Biotinylated RNA pull down assay

RNA-protein interaction was detected by RNA pulldown and immunoblotting method. 5′ end biotin labeled RNA oligoes were obtained from Bioneer Corporation. RNA oligoes at 100 pmol were immobilized on 5 μl streptavidin agarose beads. IMR-32 and SK-N-SH cells were washed with pre chilled PBS twice and gently resuspended in hypotonic buffer solution (20 mM Tris-HCl, pH 7.4, 10 mM NaCl, 3 mM MgCl_2_). Cells were lysed with NP40 (10%) by rigorous votexing. The mixture was then centrifuged at 3000 rpm at 4 °C and the nuclear extract was collected as pellet and resuspended in NP40 and quantified. RNA-bead conjugates were then incubated in nuclear extracts of IMR-32 or SK-N-SH cells at 4 °C overnight under 100 RPM rotation in a rotator. The beads were then washed 3 times and heated at 80 °C for 5 min in 1X protein loading dye. Levels of bound HuD were then detected by Western blotting. Biotinylated RNA oligo sequences (Biotin 5′- > 3′) were as follows: GRB-10_1 AUUUAUAAAUAUGCGUUUAUUUAAA, GRB-10_2 AUUUGACUUUUAUUUUUUGUAUUUA, ARL6IP1_1 GUUUUUGAAUUUAUUGCACUGAUGU, nonspecific oligo GAAAGGACUCCUUUGACAGGCAUCGG.

### Immunocytochemistry (ICC), immunohistochemistry (IHC-F), and tissue array staining

Cells and tissues were fixed with 4% paraformaldehyde (PFA) and stained with primary and secondary antibodies with DAPI. The images of the cells were captured using a confocal microscope (Carl Zeiss LSM 710) and analyzed with ZEN 2.6 software (Carl Zeiss). The antibodies used for ICC and IHC-F were listed in Tables S7 and S8, respectively.The paraffin-embedded neuroblastoma and peripheral nerve tissue array were commercially obtained (Biomax NB642a), and immunocytochemistry (IHC-F) staining was performed. The tissue array contained 27 cases of neuroblastoma, plus 5 cases of normal peripheral nerve tissue (listed in Table S9 of the supplementary methods).

### Xenograft study in athymic, nude mice

Doxy-inducible HuD shRNA expressing IMR-32 and SK-N-SH cells were cultured, harvested, and suspended in 50% matrigel (3433–001-R1, Trevigen) in PBS. Each mice was inoculated with 1 × 10^7^ cells subcutaneously in the right flank region at 5 weeks of age. The nude, athymic mice (nu/nu) obtained from DBL Korea (South Korea). Treatment started when the tumors were palpable and the mice were injected intraperitoneally with 100 μl PBS or doxycycline in PBS at 2 mg/kg body weight on alternate days, three times a week for 30 days. The experiments involving animals were approved by the Institutional Animal Care and Use Committee (IACUC) of Hallym University, Chuncheon, South Korea (approval number, Hallym2019–51) by workers trained in humane handling of laboratory animals.

### Statistical analysis

Statistical analyses were performed using GraphPad Prism 5 software (GraphPad). The analytical data are presented as mean ± SEM and compared statistically by the tests indicated. For all, *p* < 0.05 was considered as being statistically significant.

## Results

### HuD is overexpressed in neuroblastoma and is required for maintaining neuroblastoma viability

**Fig. 1:**
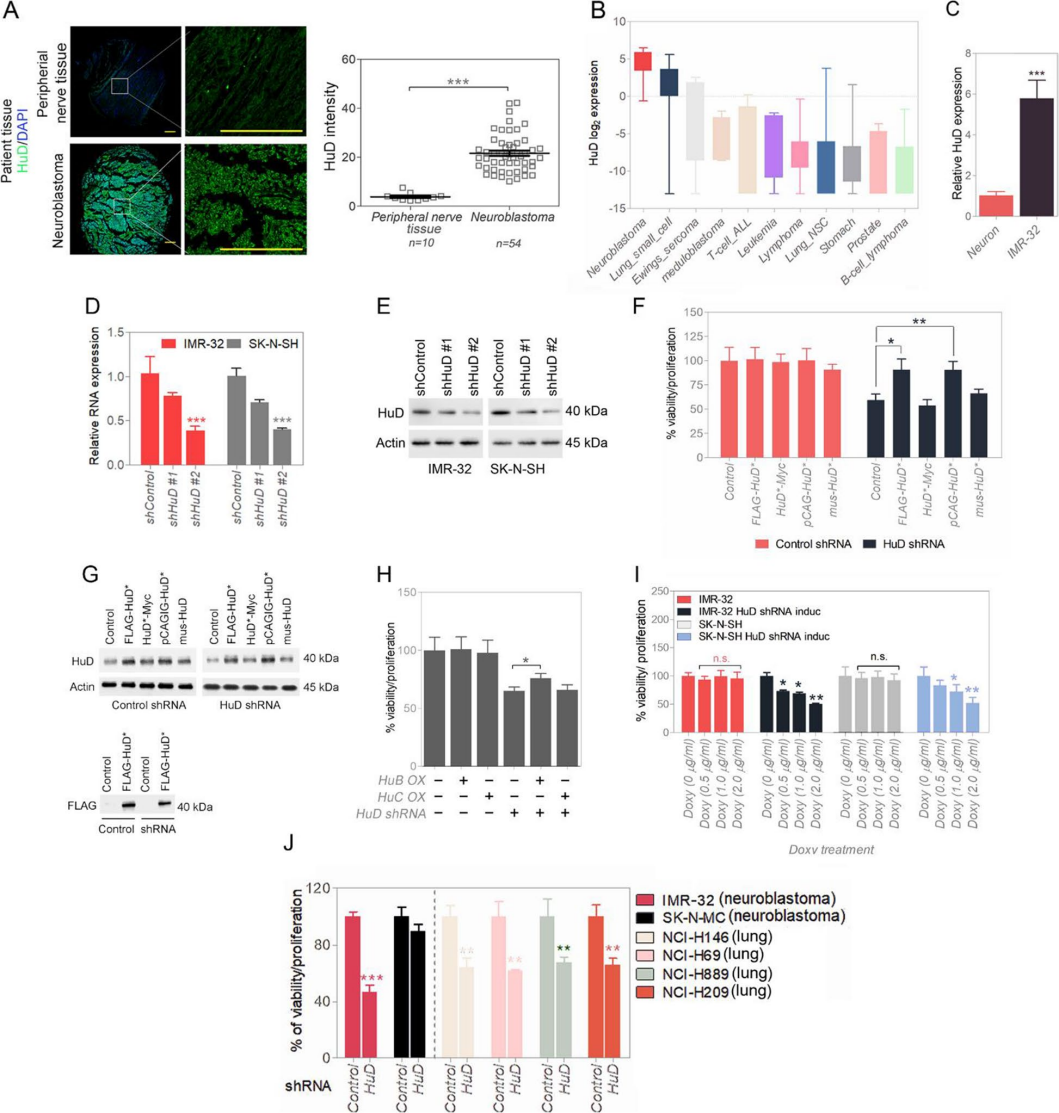
HuD is required for cancer cell survival. **A** HuD protein expression analysis in peripheral nerve tissue (normal) vs. neuroblastoma (cancer); n is the total number of samples in the patient tissue array (the array represents 27 cases of neuroblastoma, plus 5 cases of normal peripheral nerve tissue) (see Methods under “Immunocytochemistry (ICC), immunohistochemistry (IHC-F), and tissue array staining.” The array content is listed in Table S9 of the supplementary methods). Scale bar corresponds to 200 μm. Relative protein quantifications are shown (right). **B** HuD expression across different cancer cell lines obtained from CCLE; data are presented as mean ± SD. **C** Expression quantification for HuD in human neurons and neuroblastoma by RT-qPCR. **D** and **E** HuD shRNA treatment reduced HuD massage and protein expression in IMR-32 and SK-N-SH cells. Full-length blots are presented in Supplementary Fig. S9. **F** and **G** Validation of HuD shRNA and shRNA inactive HuD mutant constructs with viability and Western blotting in IMR-32 cells. Full-length blots are presented in Supplementary Fig. S9. **H** Viability assay (control or silenced HuD and/or overexpressed HuB and/or overexpressed HuC) in IMR-32 cells. **I** Validation of viability loss in shHuD inducible IMR-32 and SK-N-SH cells. **J** Viability assay comparison between HuD over-expressers - IMR-32, NCI-H146, NCI-H69, NCI-H889, NCI-H209, and low expresser - SK-N-MC (with control or silenced HuD). Data are presented as mean ± SEM; t-test: *p < 0.05, **p < 0.01, ***p < 0.001

HuD protein levels are significantly upregulated in patient neuroblastoma (NB) tissue array samples when compared with normal peripheral nerve tissue (Fig. 1A). HuD mRNA levels are also significantly higher in NB patient cohorts (Fig. S1A and B) and cell lines (Fig. 1B) compared with other patient cancer types (using the same HuD probe and changes normalized). Comparison between human neurons and human neuroblastoma IMR-32 showed relatively higher HuD signal in neuroblastoma IMR-32 cells (Fig. 1C). We sequenced HuD in IMR-32 neuroblastoma cells and found that it expressed a known wild-type transcript of HuD with no mutations at the amino acid level. With doxycycline (doxy) inducible HuD shRNA stably expressing NB cell lines (IMR-32 and SK-N-SH), we tested two shRNA HuD vectors (HuD#1 shRNA/HuD#2 shRNA) (Fig. 1D and E). Both IMR-32 and SK-N-SH overexpress HuD; IMR-32 intrinsically harbors MYCN oncogene amplification and SK-N-SH does not have MYCN amplification and does not overexpress MYCN [20]. The specificity of the shRNA for HuD was determined by addback of FLAG-HuD, silently mutated that it would not bind to shRNA HuD product. We also tested a C-terminal Myc-tagged HuD (HuD-Myc) silently mutated, but that construct was not active in the addback experiment (Fig. 1F and G). For other family members, HuB and HuC addbacks could not compensate for shRNA HuD viability loss in the test cells and only FLAG-HuD could (Fig. 1H). A culture test of the HuD silencing in these cells showed that there is reduced cell viability by 48 h post-HuD shRNA induction (Fig. 1I). With HuD shRNA treatment, all HuD high expresser neuroblastoma and small cell lung carcinoma cells had significant losses in viability/proliferation and not in low HuD-expresser SK-N-MC (Fig. 1J).

### HuD is heavily upregulated in response to stress and downregulated in presence of growth signals

**Fig. 2:**
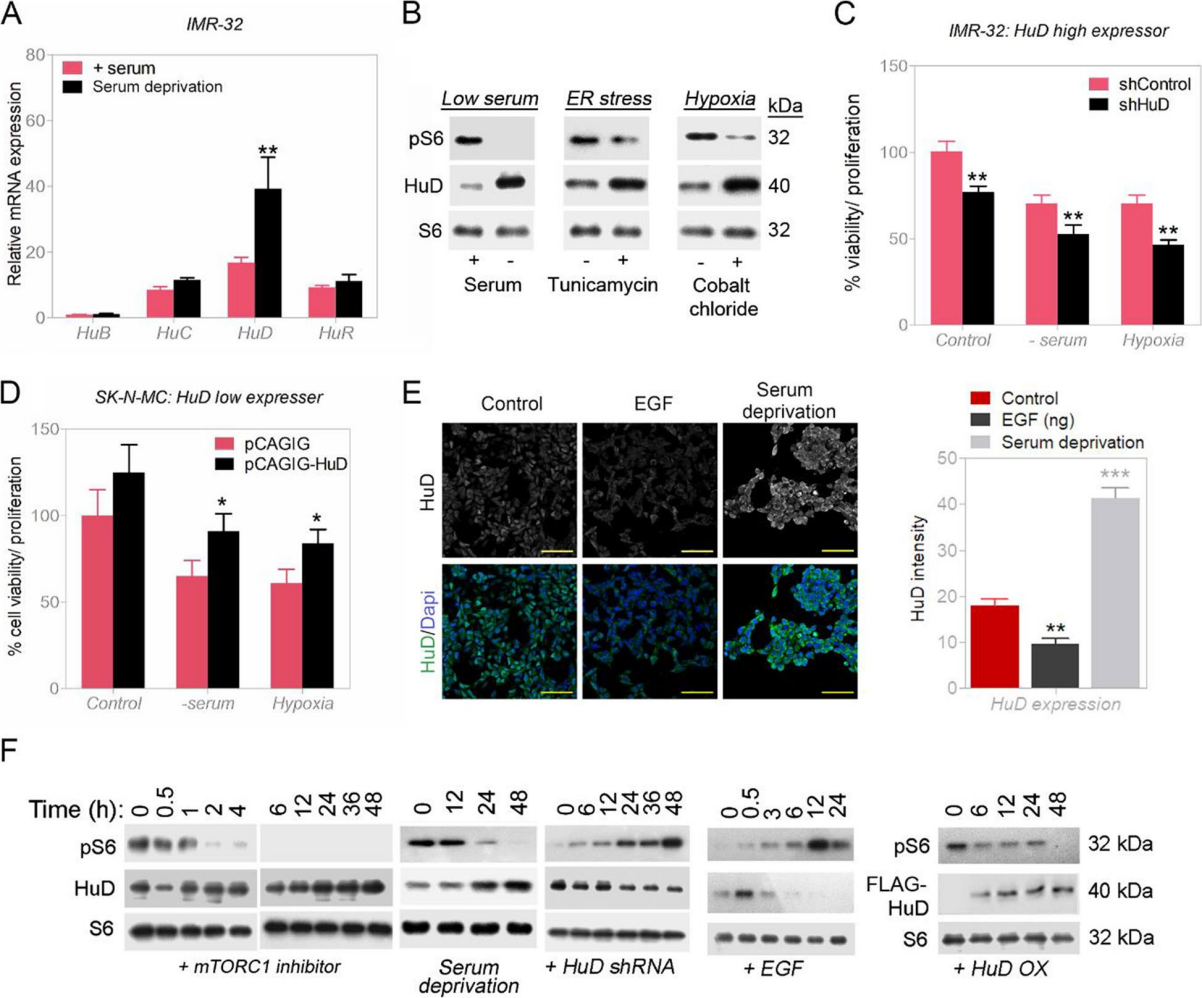
HuD levels are upregulated by stress. **A** Increase in HuD RNA levels by serum deprivation in IMR-32 cells. **B** Changes in protein HuD and pS6 levels under various forms of stress (serum starvation, ER stress and hypoxia) in IMR-32 cells by Western blot analysis. Full-length blots are presented in Supplementary Fig. S9. **C** Viability changes under stress condition in control or silenced HuD IMR-32 cells. **D** Viability changes for stressed cells (control or vector-mediated addition of HuD) in HuD low expresser SK-N-MC cells. **E** HuD protein changes in EGF supplemented and serum deprived cells condition by immunocytochemistry and their relative quantification. **F** Changes in HuD and pS6 protein following rapamycin (mTOR inhibitor), serum deprivation, silencing of HuD (HuD shRNA), vector-mediated addition of FLAG-HuD and EGF treatment by Western blot analysis. Full-length blots are presented in Supplementary Fig. S9. Data are presented as mean ± SEM; t-test: *p < 0.05, **p < 0.01, ***p < 0.001

HuD RNA and protein levels in IMR-32 NB cells increase when cells come under different conditions of stress (serum starvation, treatment with hypoxia mimic, cobalt chloride, and tunicamycin for ER stress) (Fig. 2A and B). Compared with family members, HuB, HuC and HuR, with serum starvation, the RNA message increases for HuD are many folds higher and more significant (Fig. 2A). IMR-32 cells also lose viability in presence of HuD shRNA (HuD knockdown); this is for both when under optimal growth or when stressed (Fig. 2C). Introducing additional HuD to a cell also increases viability in low HuD expresser SK-N-MC cells (HuD overexpression via transient transfection; the transfection efficiency was gauged in Fig. S1C) (Fig. 2D). In presence of an added mitogenic growth factor, in this case EGF, HuD levels are down modulated, pointing to stress/starvation as the driving force for upregulation of HuD (Fig. 2E and F).

### HuD negatively modulates mTORC1 activity

**Fig. 3:**
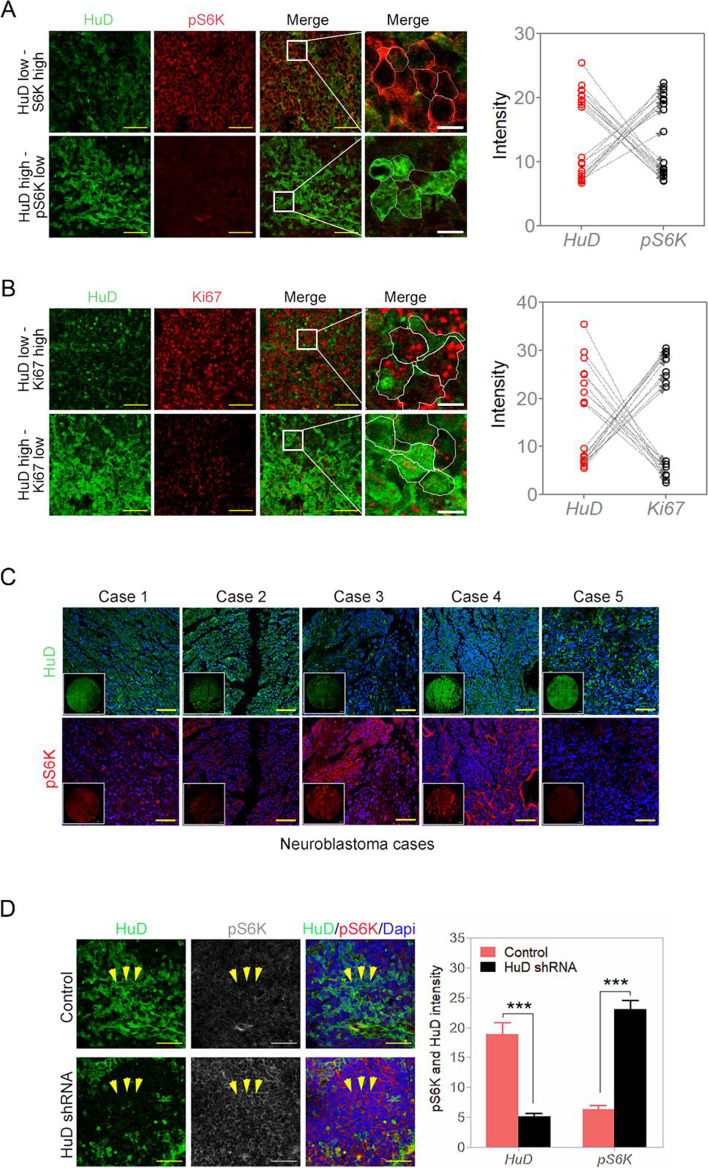
HuD and pS6 levels in neuroblastoma tumors. **A** Protein expression comparison of HuD and pS6K in IMR-32 xenograft tumor samples; yellow scale bar corresponds to 50 μm and white scale bar corresponds to 5 μm. **B** Protein expression comparison of HuD and Ki67 in IMR-32 xenograft tumor samples; yellow scale bar corresponds to 50 μm and white scale bar corresponds to 5 μm. **C** Expression comparison of HuD and pS6K in 5 neuroblastoma patients; scale bar corresponds to 200 μm. **D** Expression comparison of HuD and pS6K in IMR-32 xenograft tumor samples (control vs. silenced HuD) (refer to Fig. 8B, C and E, for the IMR-32 xenograft tumor growth, volume and weight changes). Scale bar corresponds to 50 μm and relative quantifications for “B” shown. Data are presented as mean ± SEM; t-test: *p < 0.05, **p < 0.01, ***p < 0.001

As reductions in mTORC1 activity is a hallmark of cells under stress [21, 22], we analyzed changes in HuD levels with changes in mTORC1 activity. Under stress, mTORC1 activity is diminished, according to mTORC1 activity marker pS6, while HuD levels become heavily upregulated (Fig. 2B and F). Also, inhibiting mTORC1 by rapamycin [23] leads to increased HuD levels (Fig. 2F, S2A-F). Knockdown of endogenous HuD or overexpression of FLAG-HuD led to opposite changes in pS6 levels in IMR-32 cells (Fig. 2F) and NB mouse xenograft tumor samples showed HuD level changes inversely correlating with the mTORC1 activity marker pS6K (Fig. 3A). Proliferating zones with higher Ki67 levels indicated having lower HuD levels and vice versa (Fig. 3B). These indicated HuD being upregulated in tumor areas of stress and lowered proliferation. In tumors from 5 patients, HuD “high” areas had relatively lower staining for pS6 kinase and vice versa (Fig. 3C). In mouse IMR-32 xenograft tumors, induction of HuD shRNA reduces HuD expression and increases pS6 kinase expression in tumor areas (Fig. 3D). For various NB cell lines, HuD and mTORC1 activity marker pS6 also inversely correlate and this was independent of their MYCN amplification status; MYCN is frequently amplified and overexpressed in NB [1] (Fig. S2A). mTORC1 downregulation with rapamycin had a similar GO gene profile as that of HuD-RNA binders from the RIP-SEQ analysis (Tables S10 and S11; Fig. S2C).

### HuD RNA-SEQ and knockdown profiling

**Fig. 4:**
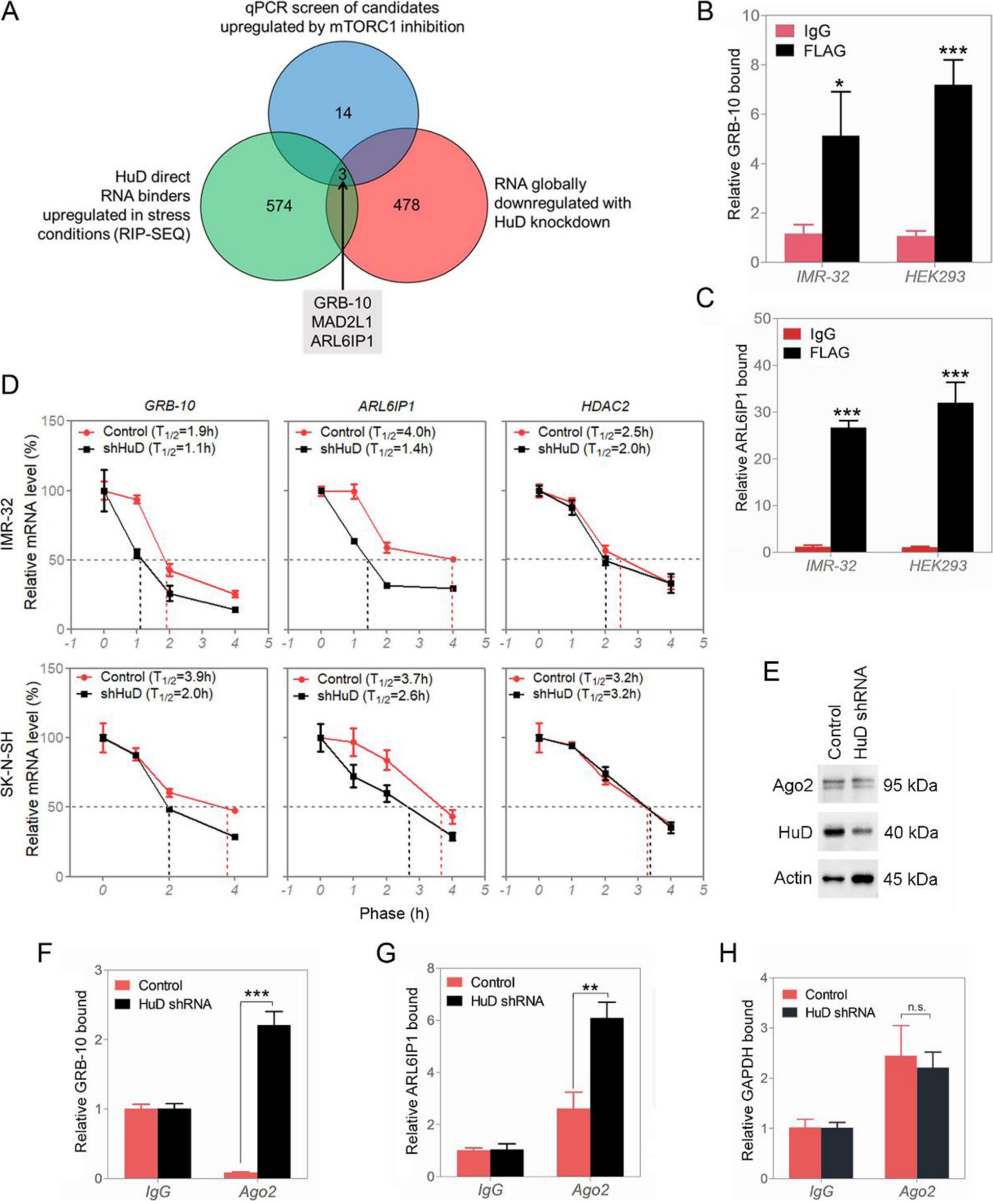
HuD interacts with and stabilizes GRB-10 and ARL6IP1 RNA transcripts in neuroblastoma cells. **A** RNA targets of HuD. **B** HuD-GRB-10 interaction by RIP/RT-qPCR in IMR-32 and HEK293 cells (control vs. FLAG-HuD-overexpressed). **C** HuD-ARL6IP1 interaction by RIP/RT-qPCR in IMR-32 and HEK293 cells (control vs. FLAG-HuD-overexpressed). **D** GRB-10, ARL6IP1 and HDAC2 RNA stability assay in IMR-32 and SK-N-SH (comparison of control and silenced HuD). **E** Western blot analysis of Ago2 and HuD in IMR-32 cells (control or silenced HuD). Full-length blots are presented in Supplementary Fig. S9. **F** Ago2 bound GRB-10; RIP/RT-qPCR in IMR-32 (control or silenced HuD). **G** Ago2 occupied ARL6IP1 mRNA by RIP/RT-qPCR in IMR-32 (control or silenced HuD). **H** Determination of Ago2 occupied GAPDH mRNA (negative control) by RIP assay followed by RT-qPCR in IMR-32 cells (control or silenced HuD). Data are presented as mean ± SEM; t test: **p < 0.01, ***p < 0.001

HuD is a known RNA binding protein in neurons [14], and to identify its RNA binding candidates in NB cells, immunoprecipitation, followed by chemical crosslinking and sequencing of RNA species (RIP-SEQ) was performed in IMR-32 cells under serum-starved conditions [24, 25]. Under the conditions of stress (serum deprivation), HuD levels in the cells are increased (Fig. 2B, E and F), recruiting key RNA binding partners to HuD that needed to be identified. RIP-SEQ identified a total of 574 RNA species as direct binders to anti-HuD antibody and not to control isotype-matched antibody from cell lysates of IMR-32 cells (Fig. 4A, Dataset S1 and S2). On the rationale that HuD binds and stabilizes key RNA substrates, HuD was silenced by shRNA for transcriptome profiling changes. This yielded a total of 478 RNA species that became downregulated with HuD shRNA treatment (Fig. 4A, Dataset S3). A total of 14 RNA candidates were common to both lists (binders to HuD and those becoming less abundant when HuD levels were lowered). This list was further reduced to ones that were under the influence of mTORC1 activity, as HuD levels increase upon treatment with mTORC1 inhibitor rapamycin (GRB-10, ARL6IP1 and Mitotic Arrest Deficient 2 Like 1 (MAD2L1)) (Fig. S3A). These candidates were confirmed by RIP-qPCR and qPCR, respectively (Fig. S3B and C). GO analysis of RIP-SEQ HuD binders implicated pathways involved in metabolism, gene expression and transport to Golgi/ ER compartment and are all related to sensing and reacting to cellular stress (Tables S10 and S11). HuD silencing affects pathways for cell division and proliferation (Table S12).

### Contrary to other HuD family members, HuD binds and stabilizes GRB-10 and ARL6IP1 mRNA

**Fig. 5:**
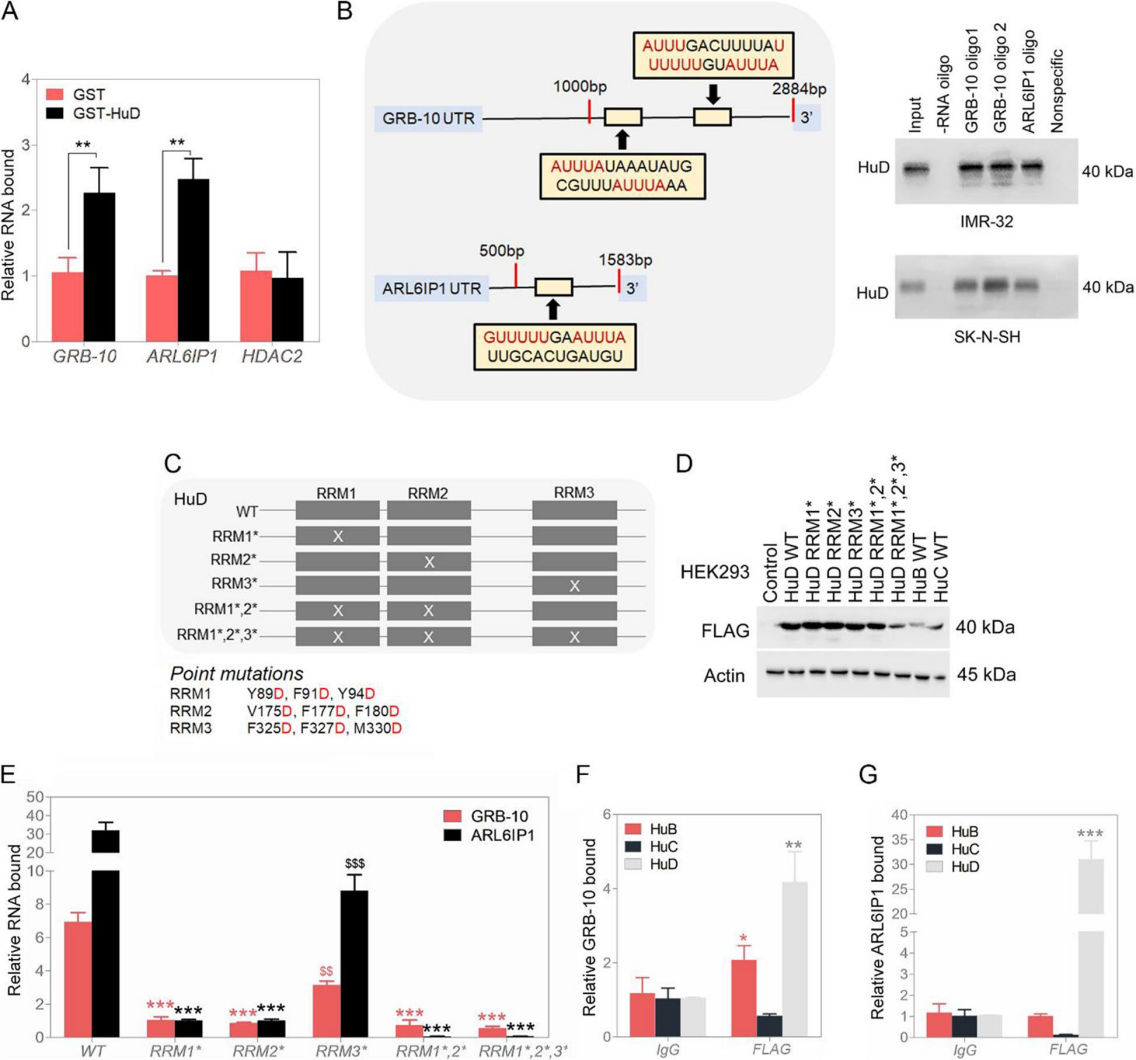
Identification of HuD protein and GRB-10 / ARL6IP1 RNA interacting domains in neuroblastoma cells. **A** GRB-10 and ARL6IP1 mRNA capture by recombinant GST-HuD; assay via RT-qPCR. **B** Biotinylated RNA oligoes specific to GRB-10 and ARL6IP1 were used to pulldown HuD protein by Western blot in IMR-32 and SK-N-SH cell lines. Nonspecific oligo and beads not coated with any oligoes served as negative controls in the capture experiments. Full-length blots are presented in Supplementary Fig. S9. **C** FLAG-HuD RNA binding domain mutants. **D** FLAG-HuD RNA binding point mutation variants expression by Western blot. Full-length blots are presented in Supplementary Fig. S9. **E** GRB-10 and ARL6IP1 RNA binding for HuD RBDs; RNAs interact with HuD and require RNA binding capable RRM domains, mainly through RRM1 and RRM2. **F** HuD-GRB-10 interaction by RIP/RT-qPCR in SK-N-DZ and SK-N-SH cells (IgG vs. anti-HuD). **G** HuD-ARL6IP1 interaction by RIP/RT-qPCR in SK-N-DZ and SK-N-SH cells (IgG vs. anti-HuD). Data are presented as mean ± SEM; t-test: t-test: *p < 0.05, **p < 0.01, ***p < 0.001 ($$*p* < 0.01, $$$*p* < 0.001 in **F**)

We pursued HuD RNA binders, GRB-10 and ARL6IP1, as identified by our Venn diagram. Binding of these targets was validated by anti-HuD antibody versus isotype control antibody immunoprecipitation, followed by qPCR in NB cell lines, IMR-32, SK-N-DZ and SK-N-SH (Fig. S4A and B). A reduction in HuD protein levels also reduces GRB-10 and ARL6IP1 cell-wide RNA levels in IMR-32 cell line, implying that HuD binds and stabilizes their mRNA (Fig. S4C). Additional control for immunoprecipitation was anti-FLAG antibody in IMR-32 and HEK293, transiently transfected with N-terminal FLAG-tagged recombinant HuD. Again GRB-10 and ARL6IP1 RNA also bound to HuD (in this case FLAG-HuD) (Fig. 4B and C). RNA stability assay with native HuD and transiently overexpressed FLAG-HuD in the assay cells was performed. Silencing of HuD via shRNA significantly reduced the relative expression of GRB-10 and ARL6IP1 and addback of HuD in form of FLAG-HuD increased relative expression of GRB-10 and ARL6IP1. After an actinomycin D block of RNA transcription, RNA levels decreased for both targets when shHuD was co-expressed, and not for the unrelated HDAC2 (Fig. 4D and S4D). HuD also protects GRB-10 and ARL6IP1 RNA from Argonaute-2 (Ago2) mediated RNA cleavage [26], not for GAPDH (Fig. 4E–H). Bacterially expressed purified N-terminal GST-HuD fusion protein also bound and increased the stability of GRB-10 and ARL6IP1 mRNA (Fig. 5A and S4E). 5′ biotin-labeled RNAs specific at the 3’UTR region for GRB-10 and ARL6IP1 were also able to pull down HuD protein from nuclear lysates and not with nonspecific oligo or oligo uncoated beads (Fig. 5B). This behavior confirmed a role for HuD in protecting its binding partners from degradation under different scenarios. Binding domains of HuD for GRB-10 and ARL6IP1 RNA binding. RRM1, 2 and 3 domains of HuD were inactivated by point mutation [27] (Fig. 5C and D). We observed that both RRM1 and RRM2 domains of HuD are required for GRB-10 and ARL6IP1 RNA binding with RRM3 mutation, showing only a partial diminution in binding (Fig. 5E).

With HuD possessing sequence similarity to HuB and HuC family members, we transiently expressed N-terminal FLAG versions of HuB, HuC and HuD for RIP experiments with anti-FLAG antibody (Fig. 5D). Compared with FLAG-HuD, GRB-10 RNA binding was also seen for FLAG-HuB and none to FLAG-HuC (Fig. 5F). For ARL6IP1 RNA, most of the binding was to FLAG-HuD (Fig. 5G). In summary, we identified HuD as binding and stabilizing two RNA substrates, GRB-10 and ARL6IP1, in neuroblastoma cells and mapped its relevant binding domains.

### HuD, by increasing GRB-10 levels, reduces mTORC1 activity

**Fig. 6:**
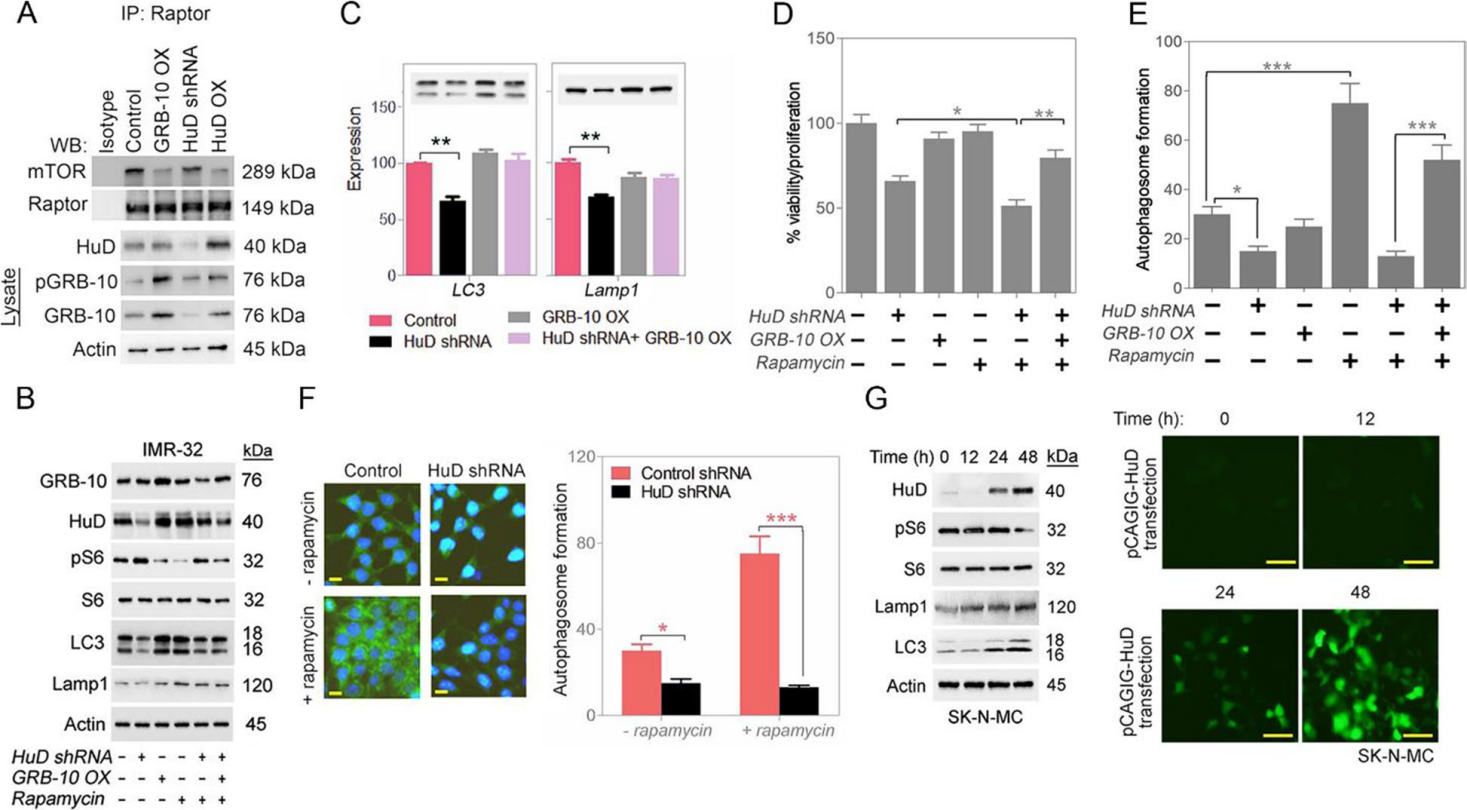
HuD and GRB-10 increase markers of autophagy in neuroblastoma cells. **A** Determination of physical binding between mTOR and Raptor (mTORC1 component) by immunoprecipitation followed by Western blotting with representative antibodies. Full-length blots are presented in Supplementary Fig. S10. **B** Western blot analysis for mTORC1 activity and autophagy markers (control vs. silenced HuD and/or overexpressed GRB-10 in absence or presence of rapamycin). Full-length blots are presented in Supplementary Fig. S10. **C** Validation of autophagy induction by Western blot and relative quantifications shown (control vs. silenced HuD and/or overexpressed GRB-10). **D** Viability assay for control or silenced HuD (HuD siRNA) and/or overexpressed GRB-10 (GRB-10 OX) and/or rapamycin in IMR-32 cells. **E** Validation of autophagolysosome formation with marker MDC for control vs. silenced HuD (HuD siRNA) and/or GRB-10 (GRB-10 OX) and/or rapamycin; relative quantifications shown. **F** Autophagolysosome formation with MDC staining (in green) (control vs. silenced HuD and/or rapamycin); scale bar corresponds to 50 μm. Relative quantifications are shown (right). **G** Western blot analysis for mTORC1 activity and autophagy markers (control or overexpressed HuD) in HuD low expresser SK-N-MC cells and transfection of pCAGIG-HuD in SK-N-MC cells at different time point. Full-length blots are presented in Supplementary Fig. S10. GFP expression was observed under fluorescent inverted microscope; scale bar corresponds to 50 μm. Data are presented as mean ± SEM; t-test: *p < 0.05, **p < 0.01, ***p < 0.001

Raptor protein regulation of mTOR kinase is via physical binding and GRB-10 has been shown to exert control [28]. Various conditions of overexpressed GRB-10, silenced HuD or overexpressed HuD were tested in IMR-32 cells with respect to Raptor and mTOR binding. GRB-10 overexpression leads to reduced binding of Raptor to mTOR (Fig. 6A) and down-modulation of mTORC1 activity in NB cell lines (IMR-32, SK-N-SH and SK-N-DZ) (Fig. 6B and S5A) [29]. Increased phosphorylated S501 GRB-10 levels lead to detachment of Raptor from mTOR complex (Fig. 6A) and induced deactivation of mTORC1 (Fig. 6B and S5A). Similar effects were seen with HuD overexpression (Fig. 6A and B) and the opposite with HuD silencing (Fig. 6A and B). These were interpreted as GRB-10, from a pool regulated by HuD, regulating mTORC1 activity.

### HuD by suppression of mTORC1 upregulates markers of autophagy

Increases in Lamp1 and LC3 markers of autophagy induction were seen when starving the cells. Direct inhibition of mTORC1 by rapamycin was used as a positive control (Fig. S2G and H). mTORC1 activity marker pS6 levels decrease with a concomitant rise in levels of HuD in the treated cells (Fig. S2G and H). Down modulation of HuD by shRNA also leads to reduced markers of autophagy (Fig. 6B−F, S5A-C) in NB cell lines (IMR-32, SK-N-SH and SK-N-DZ). Conversely, with forced overexpression of HuD, there were increased levels of autophagy markers (Fig. 6G). To further demonstrate a role for GRB-10 in inhibiting mTORC1 and activating the downstream autophagy pathway, we transiently silenced HuD and overexpressed GRB-10. Forced GRB-10 expression reduces mTORC1 activity (pS6 levels) (Fig. 6B and S5A) in NB cell lines (IMR-32, SK-N-SH and SK-N-DZ). For whether HuD-led changes in autophagy levels and mTORC1 activity are reversible when the stress conditions change, we introduced serum starvation to the cells, waited 48 h, and then supplemented them with added fresh serum and waited another 48 h. At each instance, samples of the cells were collected for Western blot analysis (Fig. S5D). Withdrawal of serum, increases HuD levels as expected along with increased autophagy and reduced pS6 levels. Serum addition to the serum starved cells reverses the HuD changes, demonstrating the reversibility of the HuD changes and its modulation of autophagy and mTORC1 levels (Fig. S5D).

### HuD increases ARL6IP1 levels, a negative regulator of apoptosis

**Fig. 7:**
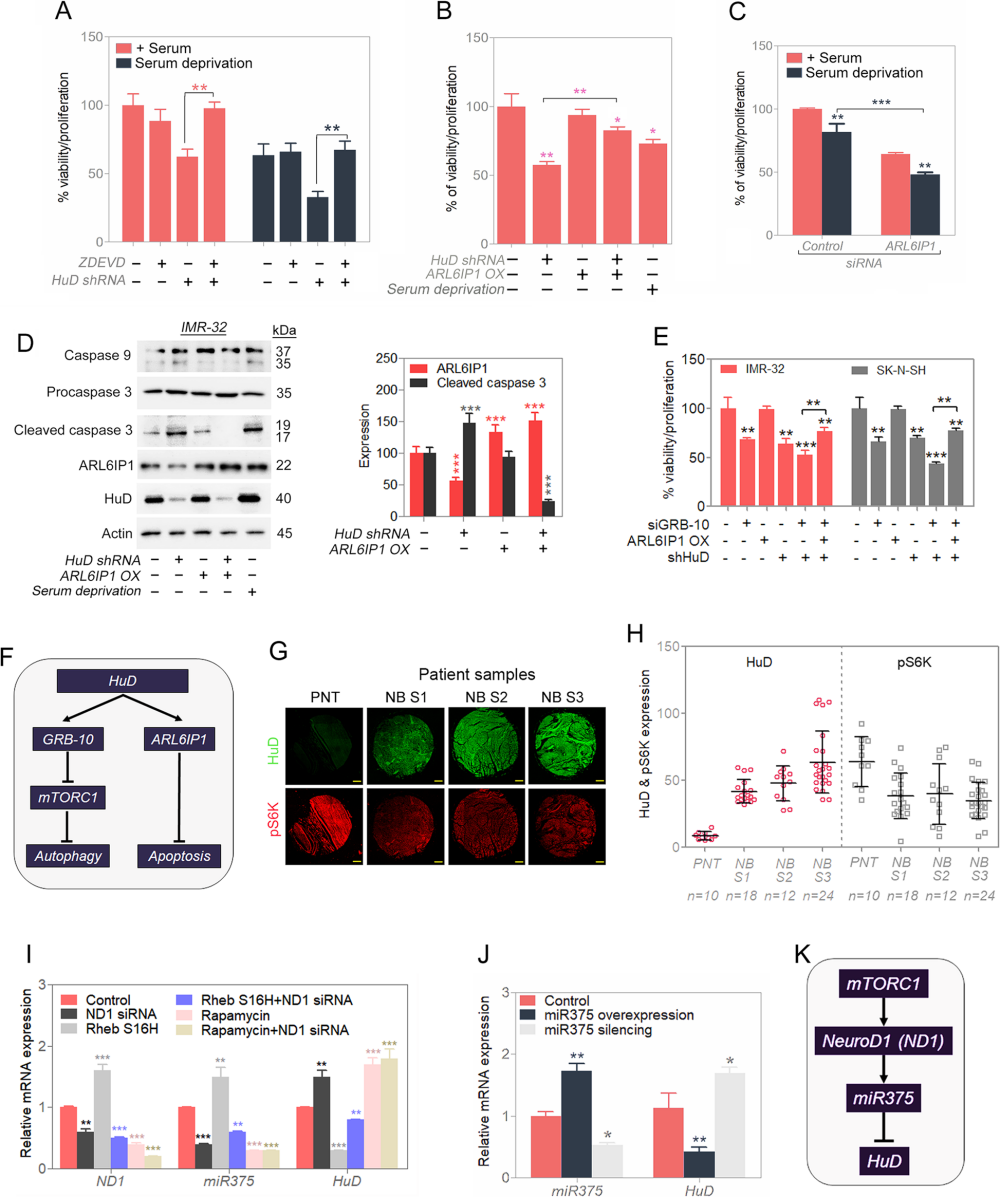
HuD produces a pro-survival signal. **A** Viability in stress condition in presence of pan-caspase inhibitor (control or silenced HuD and/or ZDEVD) in IMR-32 cells. **B** Efficiency of ARL6IP1 for controlling cell viability in IMR-32 cells (control or silenced HuD and/or overexpressed ARL6IP1). **C** Validation for efficiency of ARL6IP1 in stress condition (control or silenced ARL6IP1) in IMR-32 cells. **D** Western blot analysis for apoptosis-related protein (control or silenced HuD and/or overexpressed ARL6IP1); serum deprivation was a positive control and relative quantifications shown. Full-length blots are presented in Supplementary Fig. S10. **E** Viability assay (control or silenced HuD and/or silenced GRB-10 and/or overexpressed ARL6IP1) in IMR-32 and SK-N-SH cells. **F** Proposed schematic pathway for inhibition of cell death by HuD. **G** and **H** Immunostaining of HuD and pS6K in peripheral nerve tissue (PNT) and neuroblastoma (NB) patient of different stages, corresponding stage-wise expression quantification of HuD and pS6K levels are presented. Scale bar corresponds to 200 μm. **I** Relative mRNA expression quantified by RT-qPCR (control or silenced ND1 and/or active mTORC1 via Rheb S16H construct and/or inactive mTOR via rapamycin-25 nM) in IMR-32 cells. **J** Relative mRNA expression quantified by RT-qPCR (control or miR375 mimic or miR375 inhibitor) in IMR-32 cells. **K** Proposed schematic pathway for inhibition of HuD by mTOR. Data are presented as mean ± SEM; t-test: *p < 0.05, **p < 0.01, ***p < 0.001

HuD mediated cell viability loss was apoptosis dependent as a pan-caspase inhibitor significantly abolished the cell viability loss brought on by HuD silencing in NB cell lines (IMR-32, SK-N-SH and SK-N-DZ) (Fig. 7A and S6A). Literature shows the involvement of ER-shaping protein ARL6IP1 in preventing apoptosis by inhibiting the caspase pathway [30, 31]. We observed overexpression of ARL6IP1, the substrate of HuD, significantly protects cells against HuD silencing (Fig. 7B and S6B) in NB cell lines (IMR-32, SK-N-SH and SK-N-DZ); moreover, silencing of ARL6IP1, by itself, reduces cell viability (Fig. 7C and S6C) and its forced overexpression partially compensates for HuD knockdown (Fig. S6D). As an antiapoptotic signal, ARL6IP1 overexpression reduces active caspase 9 and 3 signals (Fig. 7D–F, S6E) in the NB cell lines. To examine whether overexpression of ARL6IP1 alone can compensate the viability loss caused by HuD and GRB-10 silencing, we overexpressed ARL6IP1 in HuD and GRB-10 silenced condition. We observed a partial recovery of cell viability by antiapoptotic ARL6IP1 overexpression in IMR-32 and SK-N-SH cells (Fig. S6F). Patient samples from 4 neuroblastoma cohorts had HuD RNA levels significantly correlate with GRB-10 and ARL6IP1 RNA levels. However, RNA message levels for ND1, MYCN, and the housekeeping controls ACTB and GAPDH transcripts did not correlate with HuD RNA message (Fig. S7). Although this was only a correlative analysis, a reading of relatively HuD high GRB-10 high ARL6IP1 high tumors may be indicative of the population of tumor types that we have been studying.

### HuD-mTORC1 interplay seems to be different in normal neurons

Given the origin of NB being from neuronal tissue [14], we compared the HuD/mTORC1 relationship in mouse cortical neurons and mouse neuroblastoma Neuro2a (N2A) cells exposed to stress (Fig. S8A). The pathways we propose seem to only apply to neuroblastoma cells and not to normal neurons. HuD levels are also significantly higher in NB patient samples than those with peripheral nerve samples (Fig. 7G and H).

### mTORC1 modulates HuD levels (via NeuroD1 and miR375)

pS6 kinase and HuD protein levels inversely correlate in patient tissue samples when compared across samples (Fig. 7G and H), and the more aggressive NB stages strongly correlate (HuD high/pS6 kinase low) (Fig. 7H). These observations were similar to those seen in mouse xenografts (Fig. 3A and D). For a possible mechanism for mTORC1-led regulation of HuD levels, we examined the role of mTORC1 dependent expression of NeuroD1 (ND1) and miR375 (Fig. 7I−K, S8B − D). Rapamycin (an inhibitor of mTORC1) and Rheb S16H overexpression (an activator of mTORC1) [23] were used to manipulate mTORC1 activity. Suppression of mTORC1 activity with rapamycin reduces ND1 and miR375 RNA levels and leads to increased HuD RNA message (Fig. 7I). Increasing the mTORC1 activity levels by Rheb S16H has the opposite effect with respect to changes in ND1, miR375 and HuD mRNA levels (Fig. 7I). Changes in viability/proliferation with respect to HuD mRNA and protein level changes were seen with miR375 mimic and inhibitor, with the mimic blocking viability/proliferation and the inhibitor promoting it (Fig. S8B). Expression changes for miR375 with respect to its mimic and inhibitor and the changes in HuD mRNA levels were confirmed by qPCR (Fig. 7J). The changes in miR375 on HuD levels were downstream of mTORC1 as rapamycin had no effect on HuD mRNA changes in presence of miR375 inhibitor (Fig. S8C). The RNA level changes in HuD were also reflected in similar changes in its protein levels (Fig. S8D). In summary, miR375 acts as an antagonist of HuD, as also previously reported [15]. mTORC1 activity positively regulates the signals for ND1 and miR375 and leads to decreased levels of HuD (both at the mRNA and protein level). Thus, regulation of HuD by mTORC1 modulates HuD levels when mTORC1 sensory network detects either optimal or survival cellular growth conditions.

### Overexpressed HuD is required for tumor generation in vivo

**Fig. 8:**
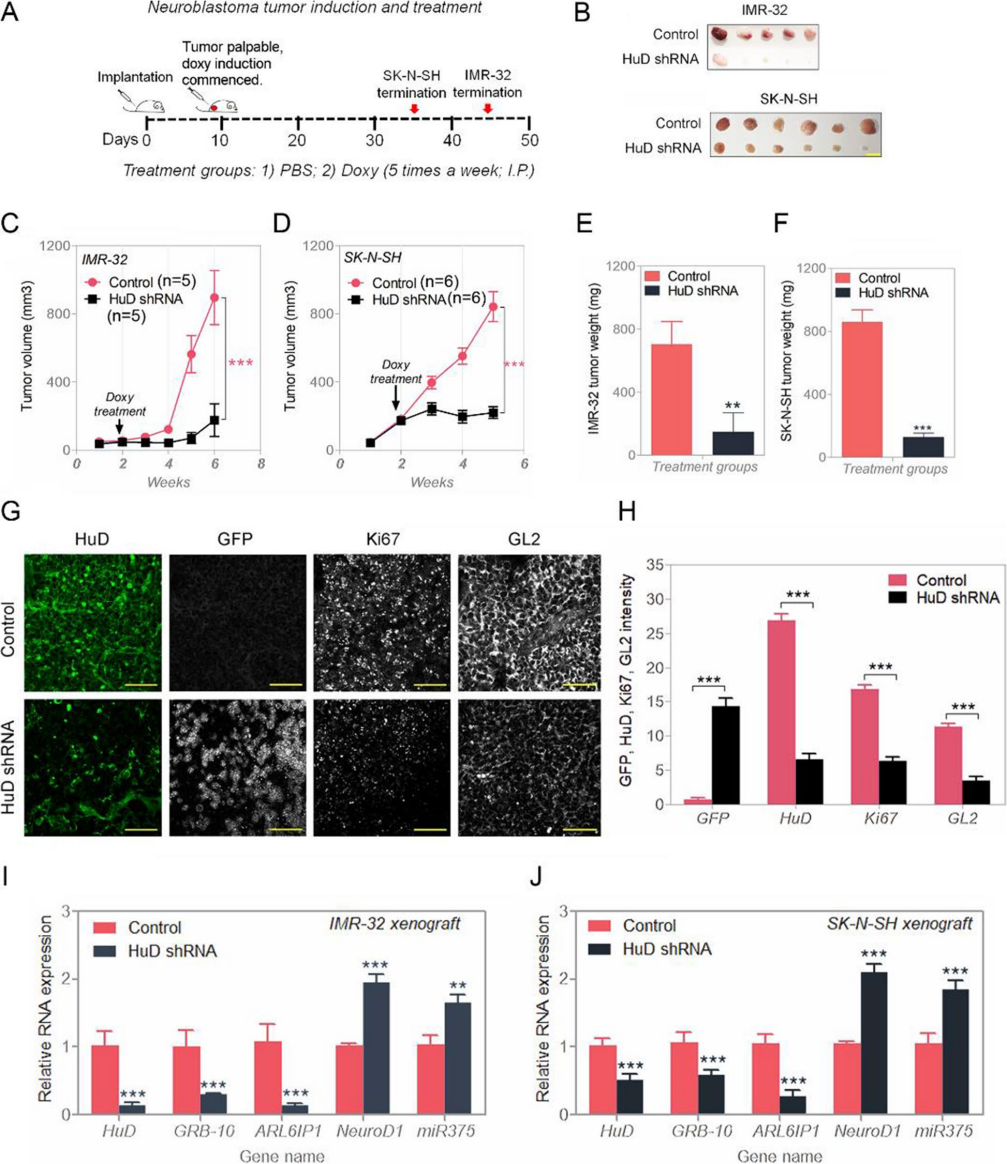
HuD is required for in vivo tumor growth. **A** Schematic representation of neuroblastoma xenograft tumor implantation and doxy induction. **B** Xenograft tumor images (control or silenced HuD). **C** and **D** Tumor volume measure for IMR-32 and SK-N-SH neuroblastoma tumor (control or silenced HuD). **E** and **F** Representative tumor weight data for IMR-32 and SK-N-SH xenograft. **G** and **H** Protein expression analysis in the tumor (control or silenced HuD). HuD, GFP (for HuD shRNA induction), Ki67 (for proliferation), and GL2 (neuroblastoma marker) expression was detected; the scale bar corresponds to 50 μm. Relative quantifications are shown (right). **I** and **J** Validation of HuD, GRB-10, ARL6IP1, NeuroD1 and miR375 signals in neuroblastoma IMR-32 and SK-N-SH xenograft (control or silenced HuD). Data are presented as mean ± SEM; t test: *p < 0.05, **p < 0.01, ***p < 0.001

Whether loss of HuD has any role in neuroblastoma tumor growth, either IMR-32 or SK-N-SH cells with inducible HuD shRNA vectors were implanted in athymic mice. shRNAs against HuD were induced by doxycycline (doxy) intraperitoneal injection after tumor sizes became palpable or they were not induced by injection with PBS for the duration of the study (Fig. 8A). Tumor growth inhibitions were observed for shRNA HuD induction in these mice in both IMR-32 and SK-N-SH xenografts (Fig. 8B–F). Post-sacrifice tumor samples confirmed induction of HuD shRNA (by upregulation of GFP as a built-in marker for doxy induction); the levels of proliferation marker Ki67 were also reduced in the induced samples; staining with GL2, a neuroblastoma marker [32], allowed identifying the tumor cells from non-tumor ones (Fig. 8G and H). From these experiments, HuD is thought to be required for tumor growth and proliferation when initially present at relatively high levels*.* Samples from NB xenograft tumors (IMR-32 and SK-N-SH) reflected a similar behavior as with the cultured cells with respect to HuD, GRB-10, ND1 and miR375: HuD silencing reduced the levels of GRB-10 and ARL6IP1 RNA and increased the expression of ND1 and miR375 (Fig. 8I and J). Therefore, HuD is required for NB tumor progression as shown by modulating mTORC1 levels and promoting autophagy and antiapoptosis.

## Discussion

We conclude that HuD is a previously undescribed promotor of autophagy via suppression of mTORC1 kinase activity. At the same time that HuD is suppressing mTORC1 activity (via GRB-10) and enhancing autophagy, we showed that HuD also promotes antiapoptosis via ARL6IP1. From these observations, we propose that in times of stress, HuD promotes autophagy and also provides an antiapoptotic signal in cancer cells. In our experiments, regulation of the RNA levels for HuD substrates GRB-10 and ARL6IP1 also occurred in mouse xenografts where silencing of HuD led to drops in the mRNA levels of GRB-10 and ARL6IP1, shown to bind and be stabilized by three versions of HuD (native and FLAG-tagged in cancer cells and purified GST-HuD protein from *E. coli*).

The RNA binding protein HuD (ELAVL4) is upregulated in a number of cancer types, particularly neuroblastoma (NB) and small cell lung cancer (SCLC), and its function in these cancers has not been well characterized. In this work, HuD seems to be required for tumor growth in NB as the inducible knockdown of HuD leads to stunted tumor growth. mTORC1 downstream substrates include 4E-BP1 and S6K1 for protein translation/signaling and ULK1 for blocking autophagy [33–36]. GRB-10 when phosphorylated causes dissociation of the mTOR positive regulator Raptor from the mTORC1 complex [28, 29]. Here, knockdown of GRB-10 and overexpression of GRB-10 modulated the activity of mTORC1, assessed by changes in the levels of phosphorylated S6 and shown by changes in the association of Raptor with mTOR. Reducing GRB-10 levels by shRNA silencing disabled HuD’s ability to modulate mTORC1 activity. We showed that HuD relieves mTORC1 inhibition on autophagy. mTORC1 reduces levels of autophagy in mammalian cells by phosphorylating ULK1 and reducing ULK1-dependent autophagy [37]. mTORC1 also blocks autophagy by phosphorylation of transcription factor TFEB, preventing its translocation to the nucleus where it would lead to the production of autophagosomal components [38]. Our work demonstrated an uptick in markers of autophagy in cases of HuD being recombinantly overexpressed.

**Fig. 9:**
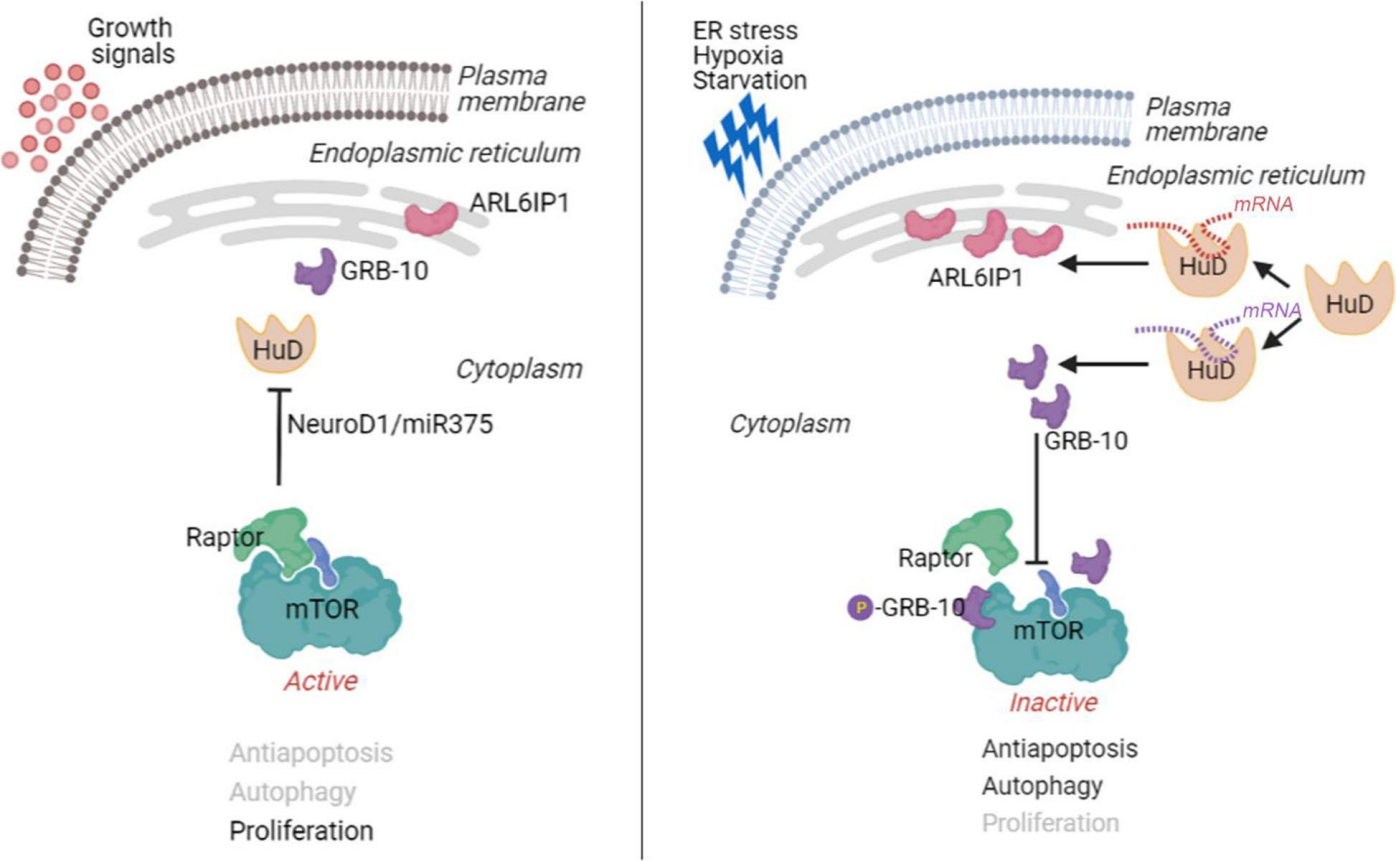
Proposed mechanism of overexpressed HuD providing pro-survival signals in neuroblastoma. Under optimal growth conditions, mTORC1 suppresses HuD expression via NeuroD1/miR375. Under various stress conditions, the mTORC1 block on HuD is lifted. The increased levels of HuD bind and stabilize RNA messages for GRB-10 and ARL6IP1. Increased levels of GRB-10 continue to limit the binding of Raptor to mTOR and keep mTOR activity at a reduced level and lift suppression on autophagy. Increased levels of ARL6IP1 provide additional pro-survival signals. ARL6IP1 is a transmembrane ER-shaping protein, functioning in preventing apoptosis by inhibiting the caspase pathway (diagram created with BioRender.com)

We showed a regulatory circuit existing for HuD, receiving input from NeuroD1 and miR375, both driven by the activity of mTORC1 [15–17]. In this sense, there may be a balancing of signals from HuD versus mTORC1, depending on inputs from the mTOR sensory network from outside of the cell such as growth factors, insulin, amino acids, nutrients and oxygen and from inside the cell for the availability of ATP [39]. See proposed schematic in Fig. 9 for modes of overexpressed HuD function in neuroblastoma cells.

There has been a report of HuD binding and stabilizing p27 Kip1 (also known as CDKN1B) mRNA and negatively impacting the patient outcome for pancreatic neuroendocrine tumor cases [40]. p27 Kip1 is a cyclin dependent kinase inhibitor, reducing CDK activity and thus blocking proliferation [41]. In our RIP-SEQ analysis, p27 Kip1 mRNA binding to native HuD in our neuroblastoma samples was not detected (SI, Dataset S1 and S2) and it may not to be part of the above reported interactions and mRNA stabilization. There has also been documentation of presence of anti-HuD autoantibodies in sera of certain neuroblastoma and small cell lung cancer (SCLC) patients and more rarely in other cancer populations [42]. The autoantibodies against SCLC-associated HuD tend to be associated with smaller tumors and patients seem to survive better [43]. As surface expressed HuD becomes isoaspartylated and is thought to become very immunogenic, there may be an immune component involved in HuD overexpressed cases [44].

The findings of the negative modulation of mTOR activity by HuD is in contrast to a recent finding that in neuronal cells, HuD contributed to mTOR functioning by upregulating many of the mTOR pathway targets including those required for protein synthesis [14]. This conundrum is in part solved by while HuD heavily downregulated mTOR kinase activity, at the same time, it compensated for reduced mTOR activity by stabilizing many of the key mTOR pathway RNA targets including those as part of the protein machinery. For survival under stress via multiple mechanisms, HuD may be contributing to the survival of these cancers.

## Conclusions

A hallmark of stress survival is mTORC1 inhibition in both normal and cancer cells. In the latter, the various inhibitory mechanisms on mTORC1 are not fully understood. Cancer cells have also advantages in resisting apoptosis. Here, we discover that the RNA binding protein HuD promotes mTORC1 inhibition during energetic and other forms of stress by promoting the mRNA levels of GRB-10. GRB-10 levels induced by HuD uncouple Raptor from mTOR kinase and lead to reduced activity of the mTORC1 complex. The reduced activity of mTORC1 then allows increased levels of pro-survival autophagy. HuD also increases the mRNA levels for ARL6IP, an apoptosis survival molecule that we show increased HuD-dependent, ARL6IP-dependent negative regulation of apoptosis. From a possible role in improving survival under stress via multiple mechanisms, HuD is thought of contributing to survival in these cancers and possibly other cancer types where it is overexpressed.

## Supplementary Information

**Additional file 1.** Supplementary information: Supplementary methods, tables and figures.

**Additional file 2. **Dataset S1_RIP SEQ full serum condition.

**Additional file 3. **Dataset S2_RIP SEQ serum starvation.

**Additional file 4. **Dataset S3_RNA SEQ HuD shRNA.

## Data Availability

All data generated or analyzed during this study are included in this published article and its supplementary information files.

## Abbreviations

ARL6IP1: ADP Ribosylation Factor Like GTPase 6 Interacting Protein 1
ELAVL4: Embryonic Lethal, Abnormal Vision, Drosophila Like RNA Binding Protein 4
eIF4A: Eukaryotic Translation Initiation Factor 4A1
FBS: Fetal Bovine Serum
FLAG tag: DYKDDDDK-tag
GRB-10: Growth Factor Receptor Bound Protein 10
GST: Glutathione S-Transferase
HuD: Hu Antigen D
MAD2L1: Mitotic Arrest Deficient 2 Like 1
MAP1B: Microtubule Associated Protein 1B
mTORC1: Mechanistic Target Of Rapamycin Complex 1
NB: Neuroblastoma
ND1: Neurogenic Differentiation Factor 1/NeuroD1
PNT: Peripheral nerve tissue
RBD: RNA-binding domains
RIP-SEQ: RNA immunoprecipitation assay and sequencing
RT-PCR: Reverse Transcriptase Polymerase Chain Reaction
