# Correction to: RNA binding protein HuD promotes autophagy and tumor stress survival by suppressing mTORC1 activity and augmenting ARL6IP1 levels

**DOI:** 10.1186/s13046-022-02275-8

**Published:** 2022-02-08

**Authors:** Kausik Bishayee, Khadija Habib, Uddin Md. Nazim, Jieun Kang, Aniko Szabo, Sung‑ Oh Huh, Ali Sadra

**Affiliations:** 1 Department of Pharmacology, College of Medicine, Institute of Natural Medicine, Hallym University, Chuncheon, South Korea; 2 Department of Anatomy, Alfaisal University, College of Medicine, Riyadh, Kingdom of Saudi Arabia

**Correction to: J Exp Clin Cancer Res 41, 18 (2022)**

**https://doi.org/10.1186/s13046-021-02203-2**

Following publication of the original article [1], the authors identified minor errors in Fig. 7, Fig. S8 and Fig. S11. Specifically:

- Fig. 7i: plotting errors in the histogram; the histogram has been corrected
- Fig. S8d: western blots (right hand side) replaced with correct blots
- Fig. S11: raw western blots presented for Fig.S8d have been replaced with correct blots

**Fig. 7:**
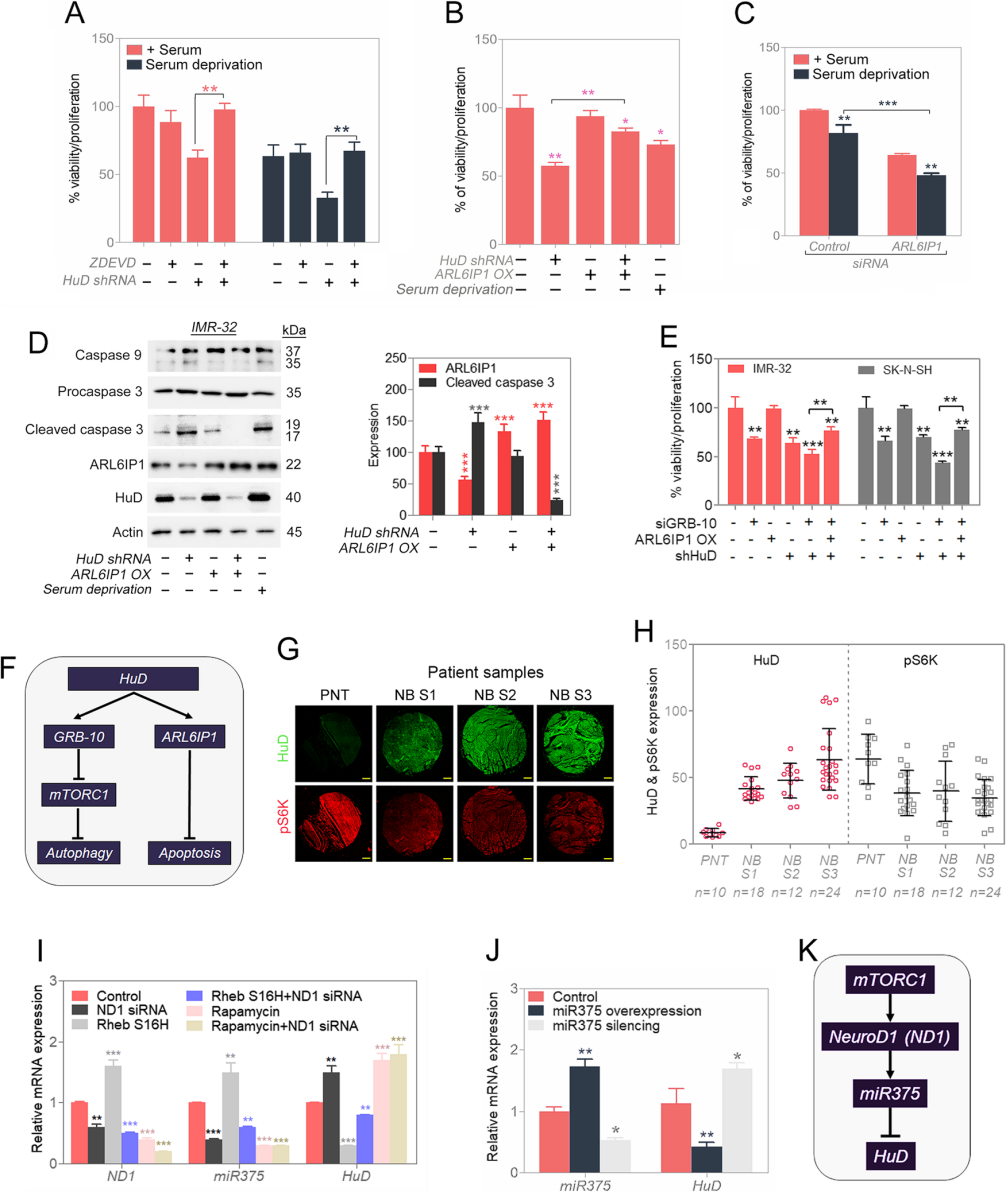
HuD produces a pro-survival signal. **A** Viability in stress condition in presence of pan-caspase inhibitor (control or silenced HuD and/or ZDEVD) in IMR-32 cells. **B** Efficiency of ARL6IP1 for controlling cell viability in IMR-32 cells (control or silenced HuD and/or overexpressed ARL6IP1). **C** Validation for efficiency of ARL6IP1 in stress condition (control or silenced ARL6IP1) in IMR-32 cells. **D** Western blot analysis for apoptosis-related protein (control or silenced HuD and/or overexpressed ARL6IP1); serum deprivation was a positive control and relative quantifications shown. Full-length blots are presented in Supplementary Fig. S10. **E** Viability assay (control or silenced HuD and/or silenced GRB-10 and/or overexpressed ARL6IP1) in IMR-32 and SK-N-SH cells. **F** Proposed schematic pathway for inhibition of cell death by HuD. **G** and **H** Immunostaining of HuD and pS6K in peripheral nerve tissue (PNT) and neuroblastoma (NB) patient of different stages, corresponding stage-wise expression quantification of HuD and pS6K levels are presented. Scale bar corresponds to 200 μm. **I** Relative mRNA expression quantified by RT-qPCR (control or silenced ND1 and/or active mTORC1 via Rheb S16H construct and/or inactive mTOR via rapamycin-25 nM) in IMR-32 cells. **J** Relative mRNA expression quantified by RT-qPCR (control or miR375 mimic or miR375 inhibitor) in IMR-32 cells. **K** Proposed schematic pathway for inhibition of HuD by mTOR. Data are presented as mean ± SEM; t-test: *p < 0.05, **p < 0.01, ***p < 0.001

The corrected figure is given here. The correction does not have any effect on the final conclusions of the paper. The original article has been corrected.

## Supplementary Information

**Additional file 1: Fig. S8** mTORC1’s inhibitory effect on HuD. **A**. Comparison between mouse neuroblastoma and neurons under stress; Akt-mTOR pathway examined by Western blot assay. Full-length blots are presented in Supplementary Figure S11. **B.** Viability assay (control or miR375 mimic or miR375 inhibitor) in IMR-32 cells. **C.** Relative mRNA expression quantified by RT-qPCR (control or miR375 inhibitor and/or active mTOR via Rheb S16H construct and/or inactive mTOR via rapamycin-25 nM) in IMR-32 cells. **D.** Western blot analysis of HuD and pS6 in IMR-32 cells. Full-length blots are presented in Supplementary Figure S11. Data are presented as mean ± SEM; t test: *p < 0.05, **p < 0.01, ***p < 0.001. **Fig. S11** Raw Western blot images for Fig. S5, S6 and S8.
