# Supplementary material for: Correction to: RNA binding protein HuD promotes autophagy and tumor stress survival by suppressing mTORC1 activity and augmenting ARL6IP1 levels
Source: J Exp Clin Cancer Res. 2022 Feb 8;41:52. doi: 10.1186/s13046-022-02275-8 (PMC8822731; doi:10.1186/s13046-022-02275-8)
Supplement: Supplementary file 1 — Additional file 1: Fig. S8 mTORC1’s inhibitory effect on HuD. A. Comparison between mouse neuroblastoma and neurons under stress; Akt-mTOR pathway examined by Western blot assay. Full-length blots are presented in Supplementary Figure S11. B. Viability assay (control or miR375 mimic or miR375 inhibitor) in IMR-32 cells. C. Relative mRNA expression quantified by RT-qPCR (control or miR375 inhibitor and/or active mTOR via Rheb S16H construct and/or inactive mTOR via rapamycin-25 nM) in IMR-32 cells. D. Western blot analysis of HuD and pS6 in IMR-32 cells. Full-length blots are presented in Supplementary Figure S11. Data are presented as mean ± SEM; t test: *p < 0.05, **p < 0.01, ***p < 0.001. Fig. S11 Raw Western blot images for Fig. S5, S6 and S8. [file 13046_2022_2275_MOESM1_ESM.docx]

**Supplementary information**

**Supplementary figures and legends**

**Fig. S1**

**
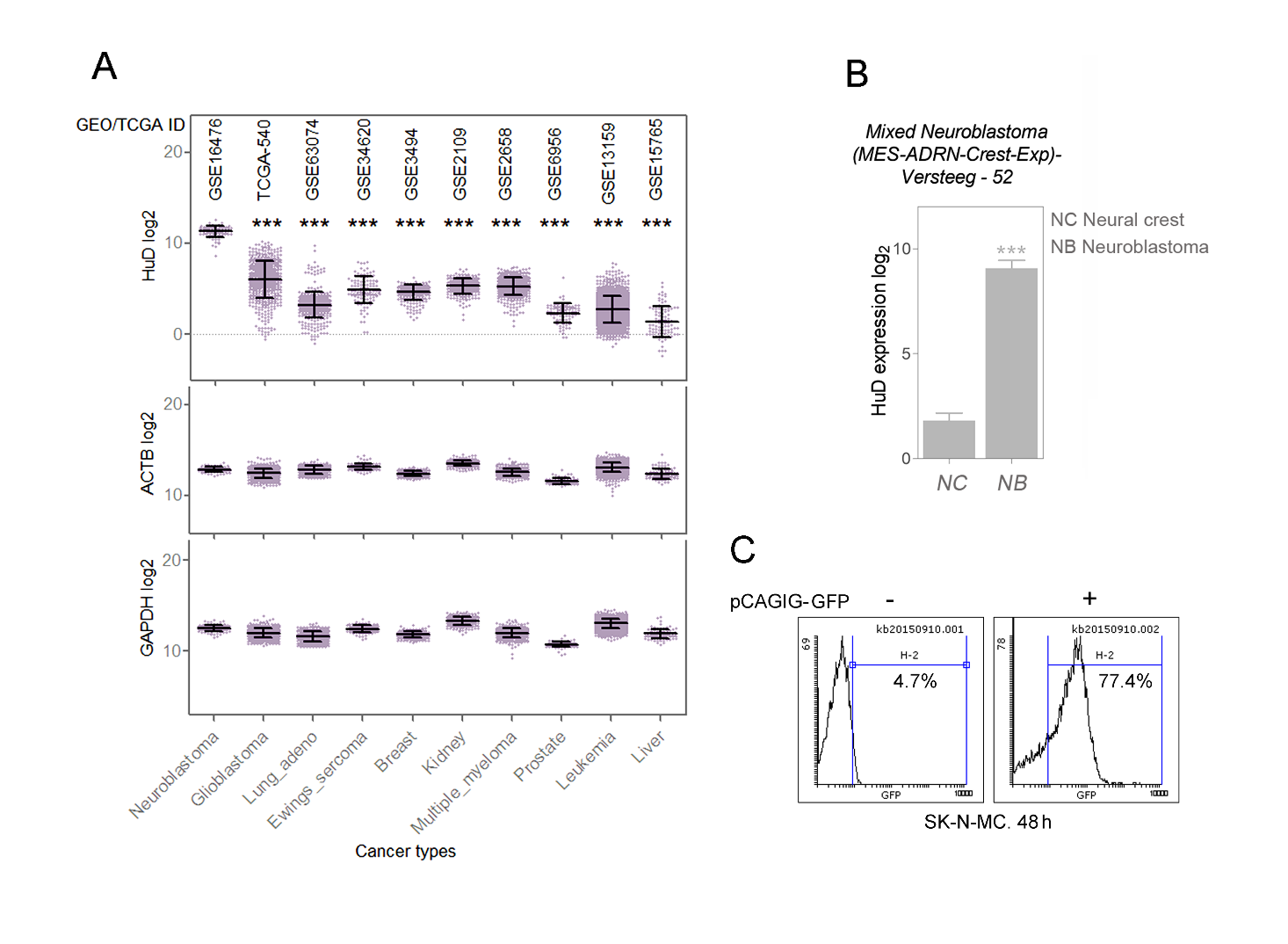
**

**Fig. S1** HuD expression in neuroblastoma is required for maintaining viability. **A.** HuD expression for cancer patients obtained from R2. **B.** HuD expression comparison between normal neural crest and neuroblastoma. **C.** SK-N-MC cells were transfected with or without pCAGIG (GFP) vector; the transfection efficacy was about 72% at 48 h. Data are presented as mean ± SEM; t test: *p < 0.05, **p < 0.01, ***p < 0.001.

**Fig. S2**

**
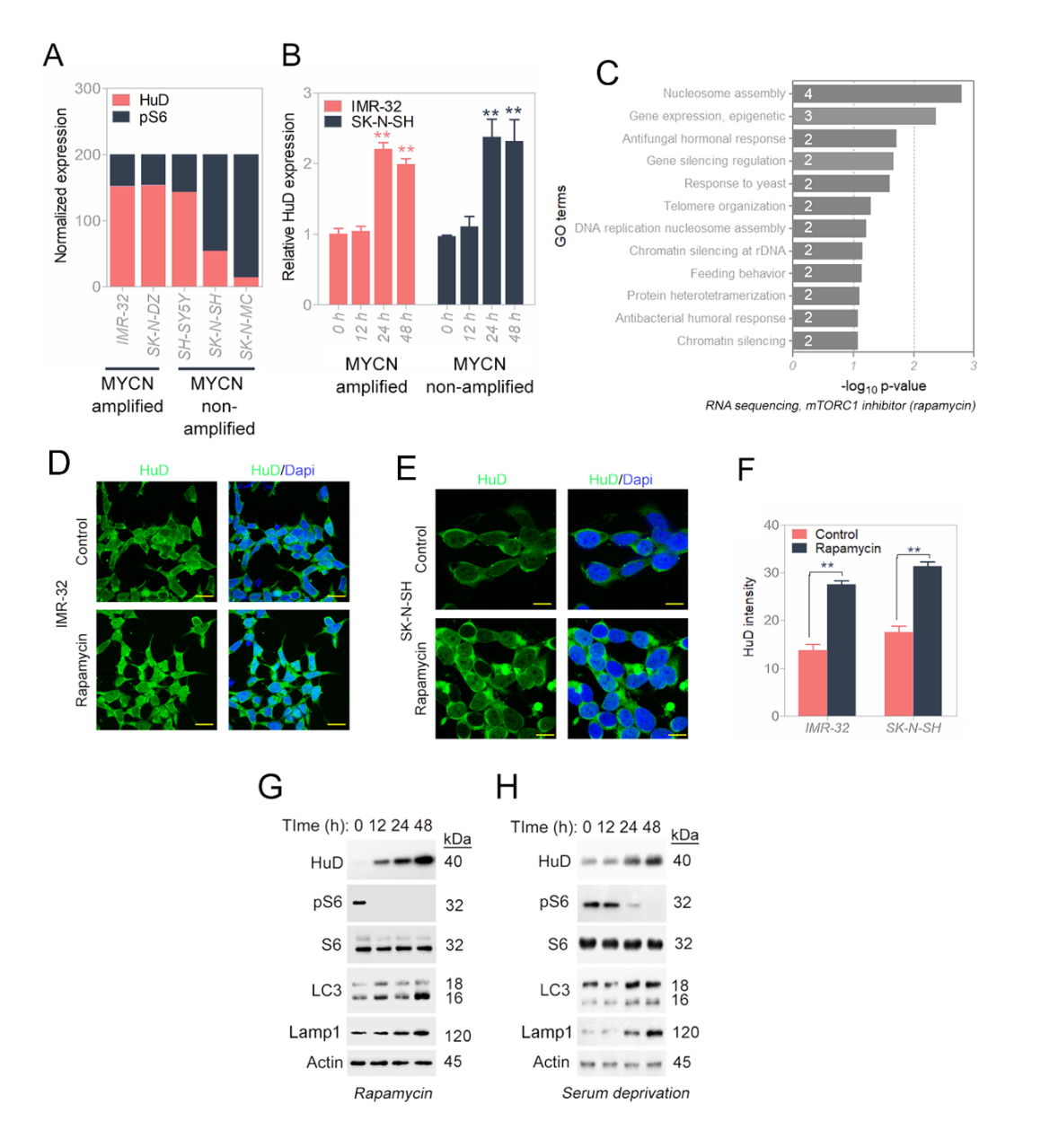
**

**Fig. S2** mTORC1 inhibition leads to increased HuD and autophagy levels. **A.** Expression analysis of HuD and pS6 in neuroblastoma cells; data are plotted as histogram. **B.** Relative HuD signal quantified by RT-qPCR (control or rapamycin-25 nM) in neuroblastoma cells. **C.** Top enriched Gene Ontology terms for transcripts changed post mTORC1 deactivation are related to gene expression and silencing response. **D** and **E.** IMR-32 and SK-N-SH cultures (control or rapamycin-25 nM) were immunostained for HuD; scale bar corresponds to 10 μm. **F.** Relative quantification of HuD levels in “C” and “D”. **G.** Western blot analysis of autophagy-related protein expression changes for control or rapamycin-25 nM. **H.** Western blot analysis of autophagy-related protein expression changes for control or serum starvation. Full-length blots are presented in Supplementary Figure S11. Data are presented as mean ± SEM; t test: *p < 0.05, **p < 0.01, ***p < 0.001.

**Fig. S3**

**
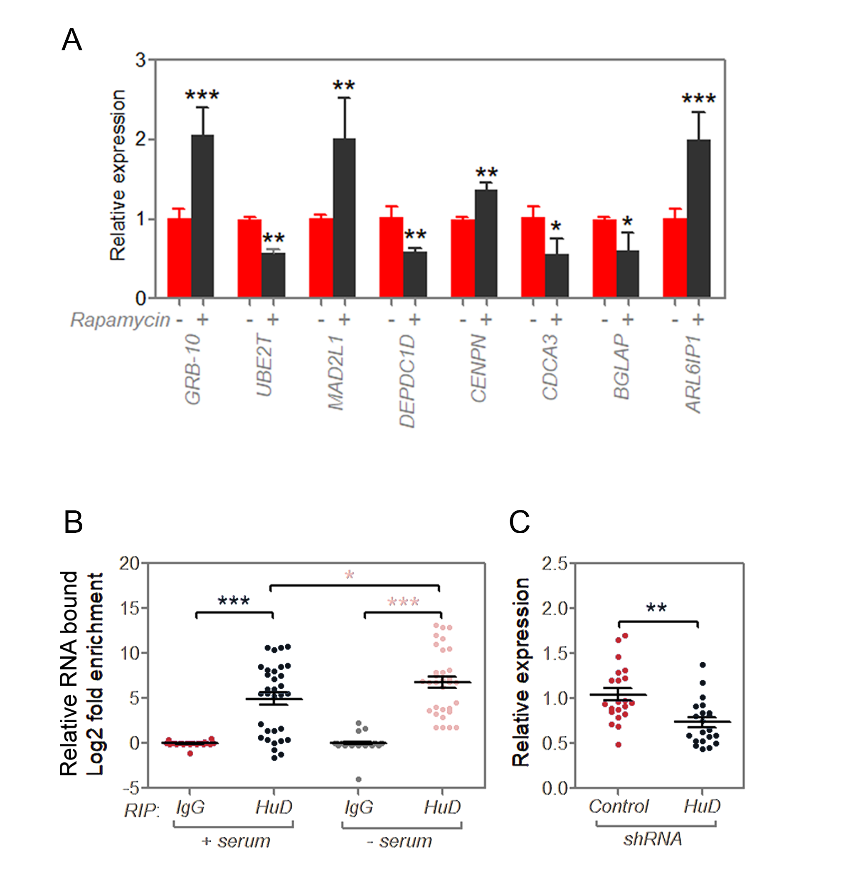
**

**Fig. S3** Defining the HuD substrates. **A.** Relative signal of HuD binders quantified by RT-qPCR (control or rapamycin-25 nM) in IMR-32 cells. **B.** Validation of HuD and its top target mRNAs by RIP followed by RT-qPCR in IMR-32 cells (w/o stress). **C.** Validation of HuD’s top target mRNAs in IMR-32 cell (control or silenced HuD). Data are presented as mean ± SEM; t test: *p < 0.05, **p < 0.01, ***p < 0.001.

**Fig. S4**

**
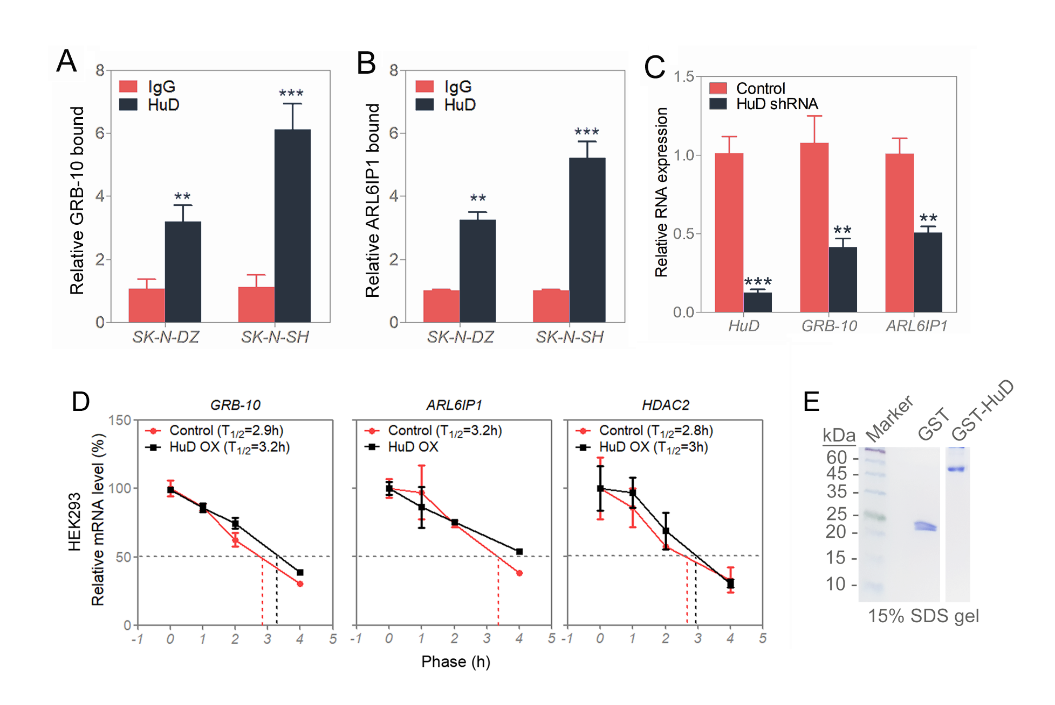
**

**Fig. S4** HuD RNA-binding domains are required for GRB-10 and ARL6IP1 RNA interaction. **A.** Validation of HuD-GRB-10 interaction by RIP followed by RT-qPCR in SK-N-DZ and SK-N-SH cells (IgG or HuD). **B.** Validation of HuD-ARL6IP1 interaction by RIP assay followed by RT-qPCR in SK-N-DZ and SK-N-SH cells (IgG or HuD). **C.** Relative RNA expression of HuD, GRB-10 and ARL6IP1 in IMR-32 cells (control or silenced HuD). **D.** GRB-10, ARL6IP1 and HDAC2 RNA stability assay in HEK293 cells (comparison of control and overexpressed HuD). **E.** Representative Coomassie /SDS-PAGE gel image of recombinant GST. Data are presented as mean ± SEM; t test: **p < 0.01, ***p < 0.001.

**Fig. S5**

**
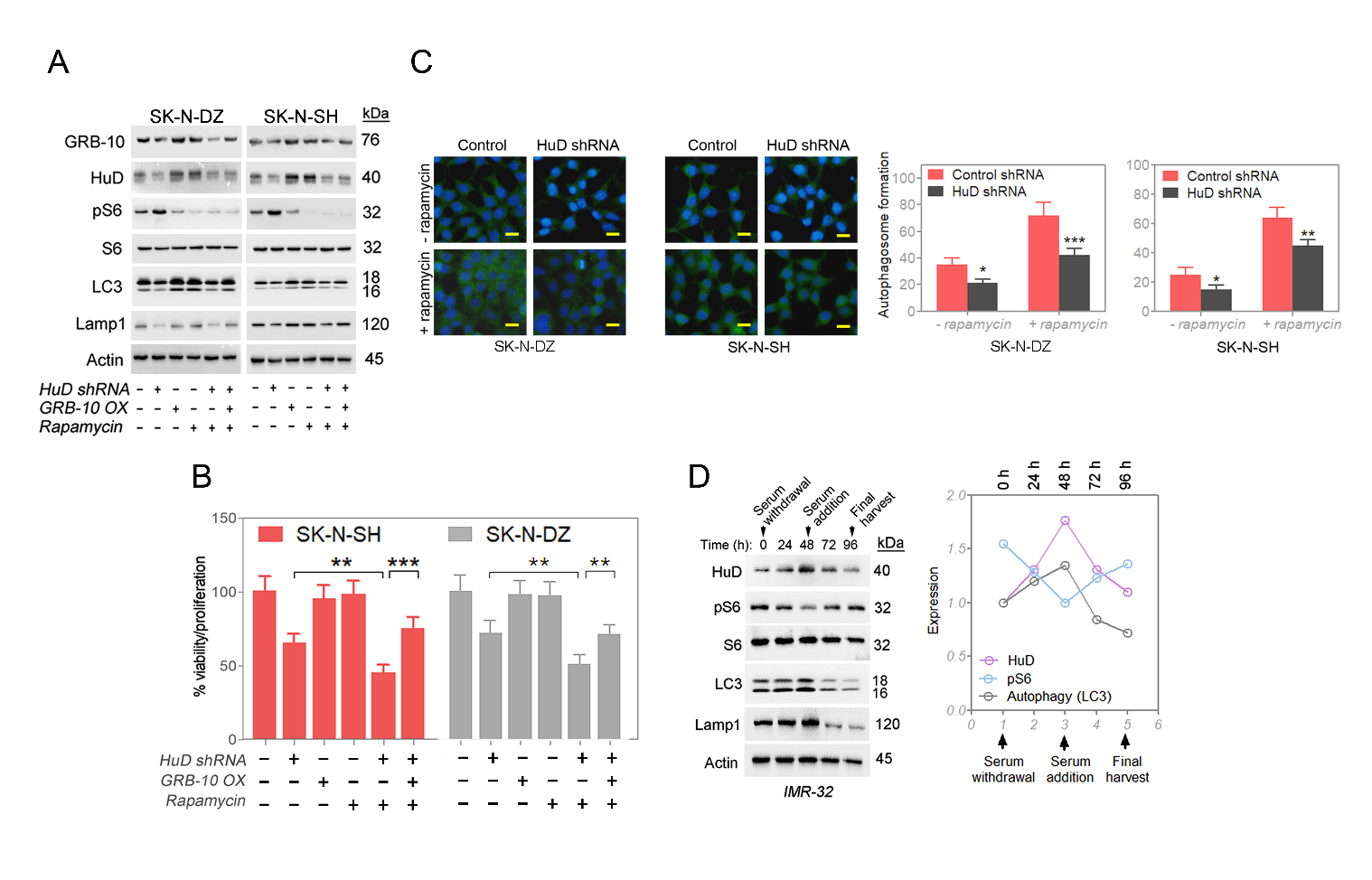
**

**Fig. S5** In two additional NB cell lines (SK-N-DZ and SK-N-SH): **A.** Western blot analysis for mTORC1 activity and autophagy markers (control vs. silenced HuD and/or overexpressed GRB-10 in absence or presence of rapamycin). Full-length blots are presented in Supplementary Figure S11. **B.** Viability assay for control or silenced HuD (HuD siRNA) and/or overexpressed GRB-10 (GRB-10 OX) and/or rapamycin in SK-N-MC and SK-N-DZ cells. **C.** Autophagolysosome formation with MDC staining (in green) in SK-N-MC and SK-N-DZ cells (control vs. silenced HuD and/or rapamycin); scale bar corresponds to 50 μm. Relative quantifications are shown (right). Reversibility characteristics of HuD and mTORC1 activity. **D.** Western blot analysis for reversibility characteristics of HuD and mTORC1 activity (pS6) and autophagy-related protein due to serum starvation and followed by serum supplementation. Full-length blots are presented in Supplementary Figure S11. The line diagram represents the expression trends for HuD, pS6 and autophagy at different time points. Data are presented as mean ± SEM; t test: **p < 0.01.

**Fig. S6**

**
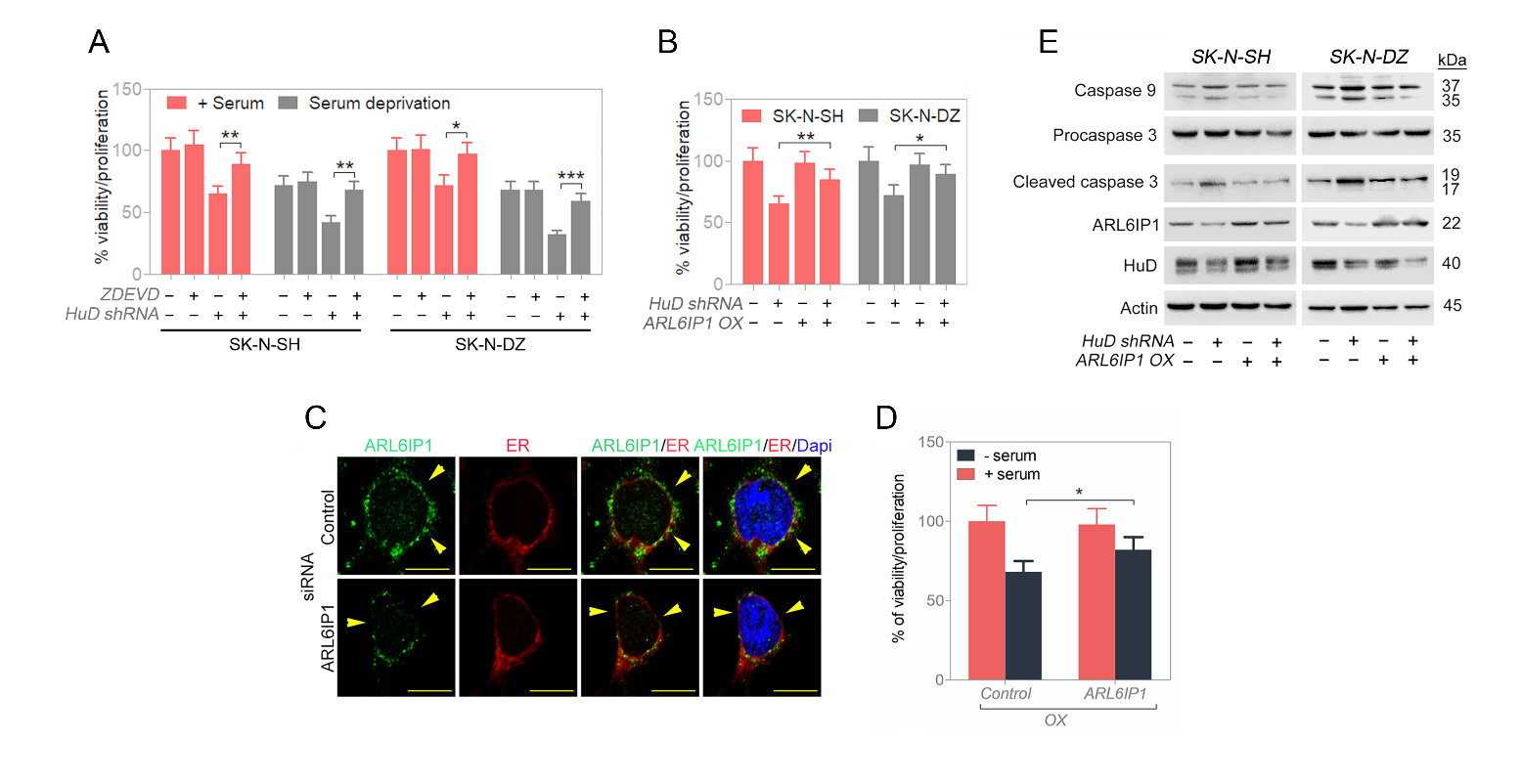
**

**Fig. S6** In two additional NB cell lines (SK-N-DZ and SK-N-SH), ARL6IP1 exhibits antiapoptotic characteristics. **A.** Viability in stress condition in presence of pan-caspase inhibitor (control or silenced HuD and/or ZDEVD). **B.** Efficiency of ARL6IP1 for controlling cell viability in cells (control or silenced HuD and/or overexpressed ARL6IP1). **C.** Localization of ARL6IP1 at ER compartment (control or silenced ARL6IP1). **D.** Viability assay in normal and serum starvation condition (control or overexpressed ARL6IP1) in IMR-32 cells. **E.** In two additional NB cell lines (SK-N-DZ and SK-N-SH), Western blot analysis for apoptosis-related protein (control or silenced HuD and/or overexpressed ARL6IP1); serum deprivation was a positive control and relative quantifications shown. Full-length blots are presented in Supplementary Figure S11. Scale bar corresponds to 10 μm. Data are presented as mean ± SEM; t test: *p < 0.05, **p < 0.01.

**Fig. S7**

**
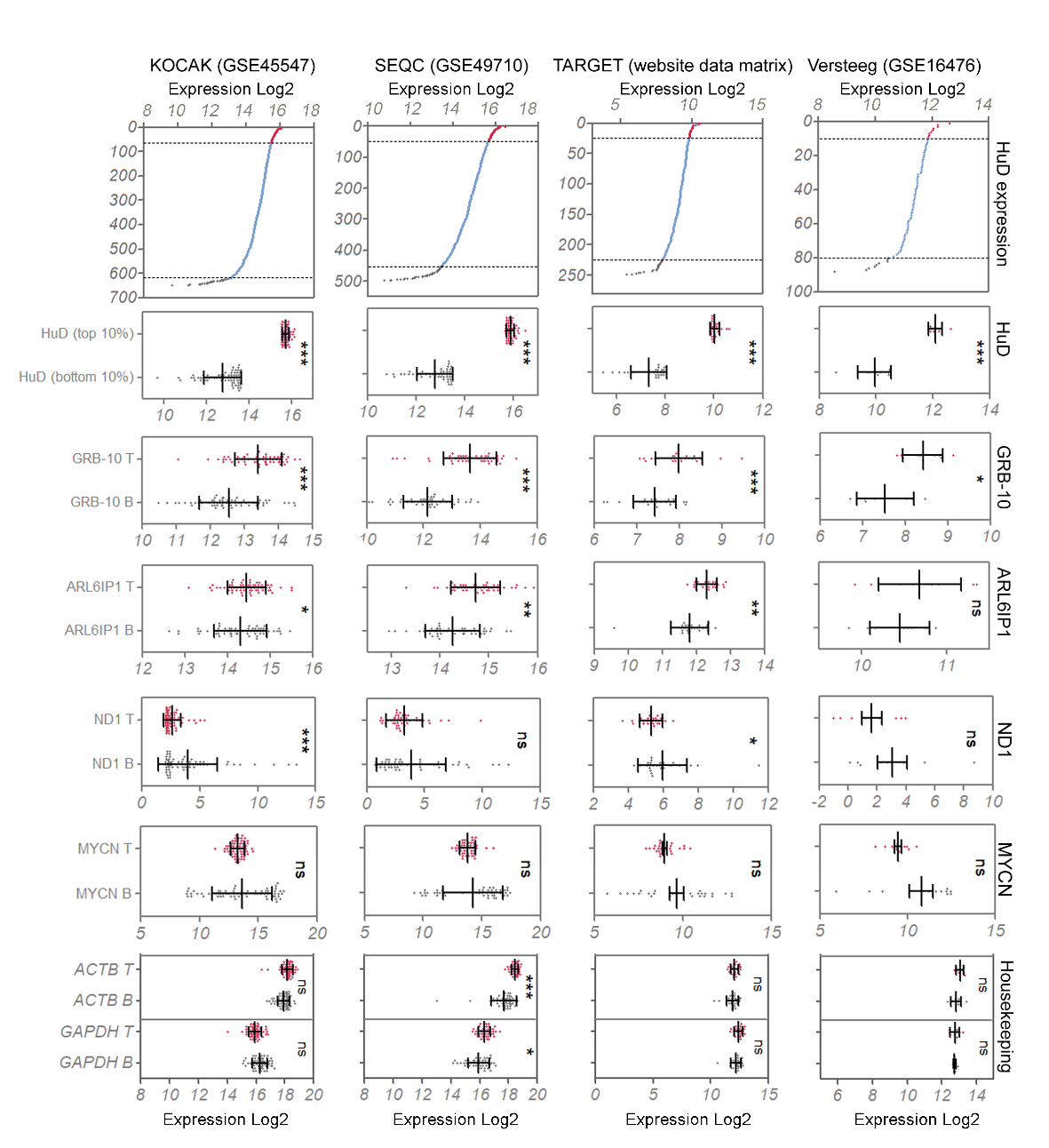
**

**Fig. S7** HuD, GRB-10 and ARL6IP1 RNA messages positively correlate in neuroblastoma patient samples. HuD expression in neuroblastoma patient cohorts was obtained from R2 and plotted; the top and bottom 10% population of HuD expressers were then selected for analyzing relative GRB-10, ARL6IP1, NeuroD1 (ND1) and MYCN signals; data are shown as log2 fold change. Data are presented as mean ± SEM; t test: *p < 0.05, **p < 0.01, ***p < 0.001.

**Fig. S8**


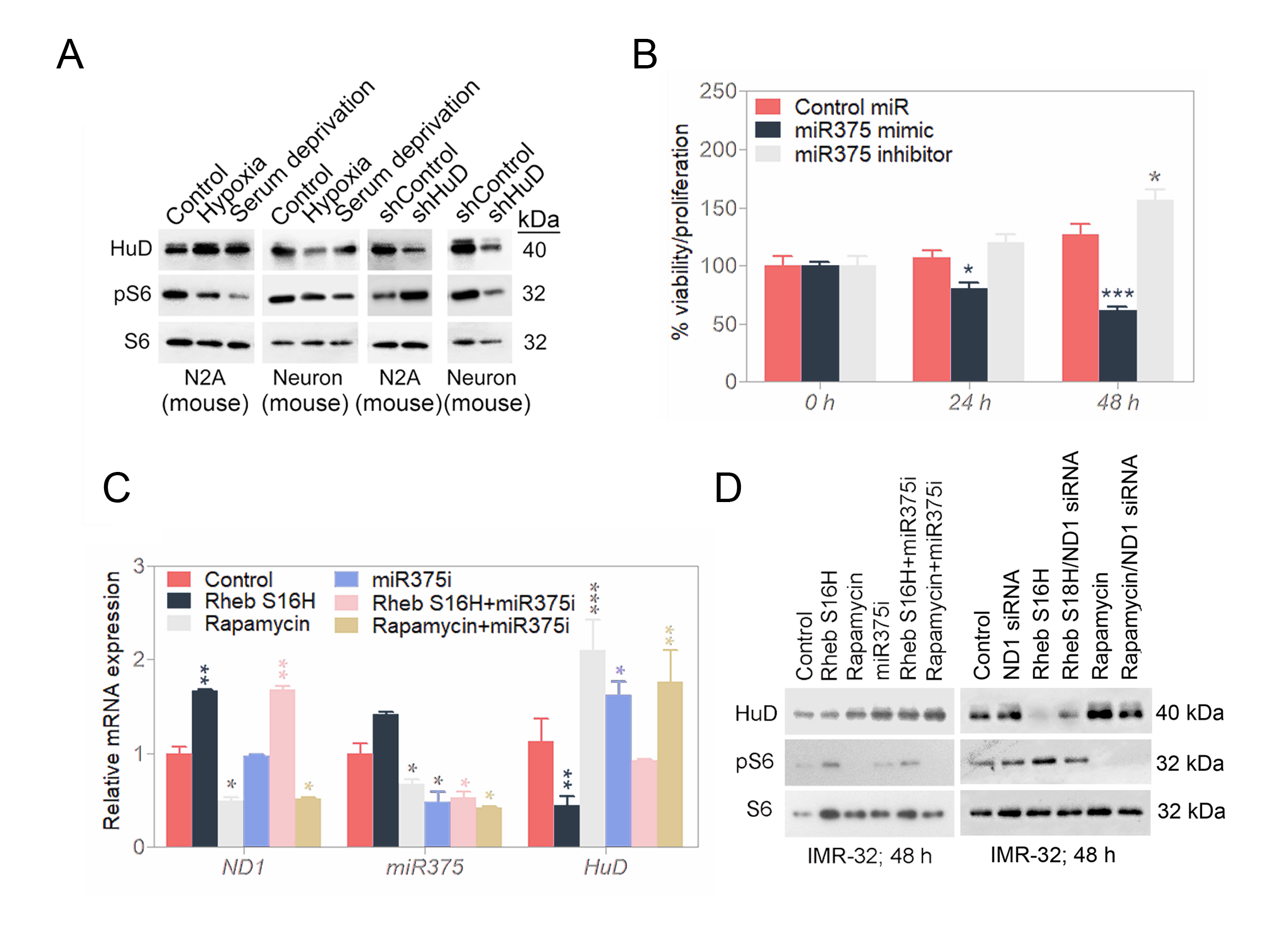


**Fig. S8** mTORC1’s inhibitory effect on HuD. **A**. Comparison between mouse neuroblastoma and neurons under stress; Akt-mTOR pathway examined by Western blot assay. Full-length blots are presented in Supplementary Figure S11. **B.** Viability assay (control or miR375 mimic or miR375 inhibitor) in IMR-32 cells. **C.** Relative mRNA expression quantified by RT-qPCR (control or miR375 inhibitor and/or active mTOR via Rheb S16H construct and/or inactive mTOR via rapamycin-25 nM) in IMR-32 cells. **D.** Western blot analysis of HuD and pS6 in IMR-32 cells. Full-length blots are presented in Supplementary Figure S11. Data are presented as mean ± SEM; t test: *p < 0.05, **p < 0.01, ***p < 0.001.

**Fig. S9**


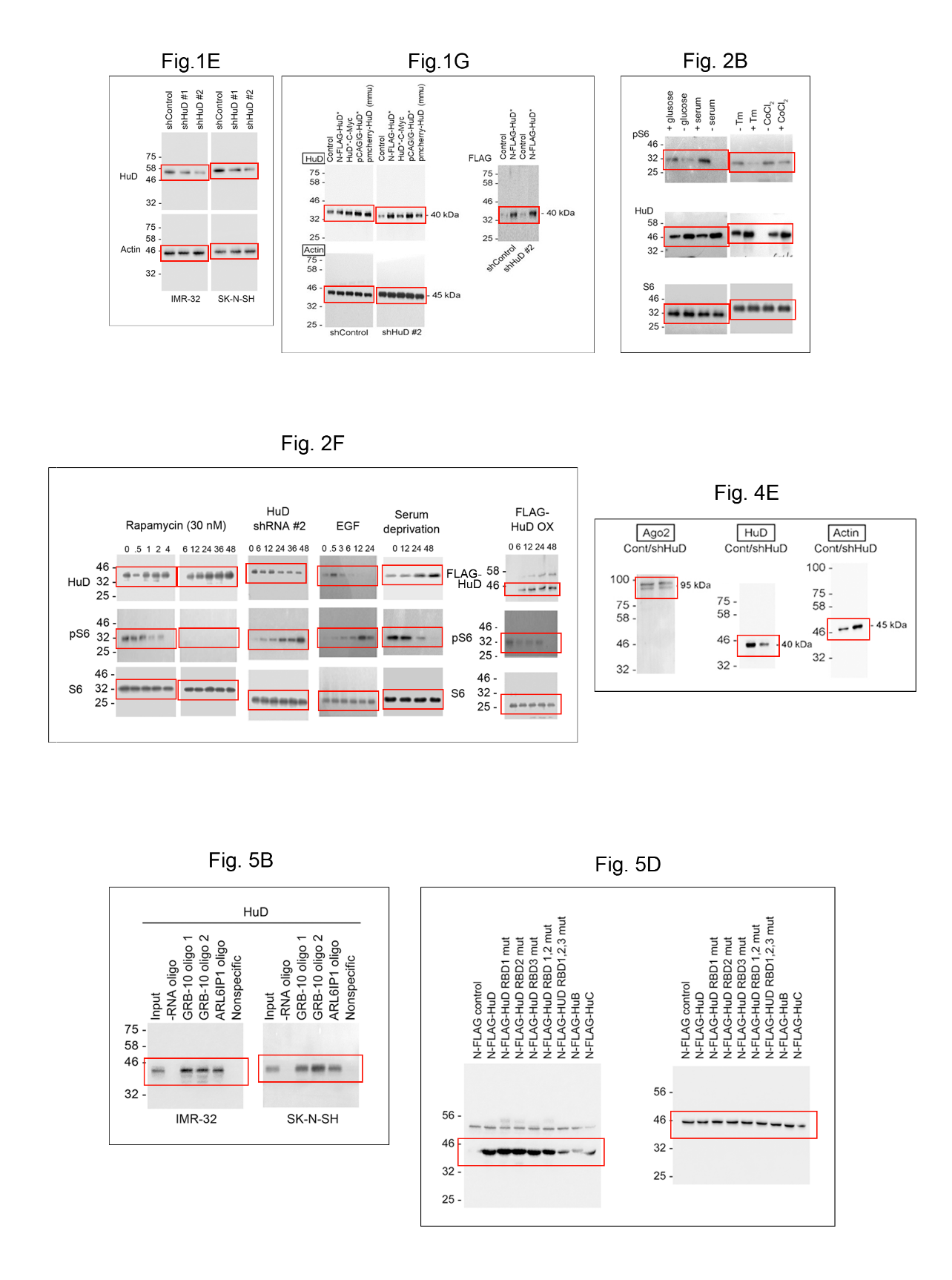


**Fig. S9** Raw Western blot images for Fig. 1, 2, 4 and 5

**Fig. S10**


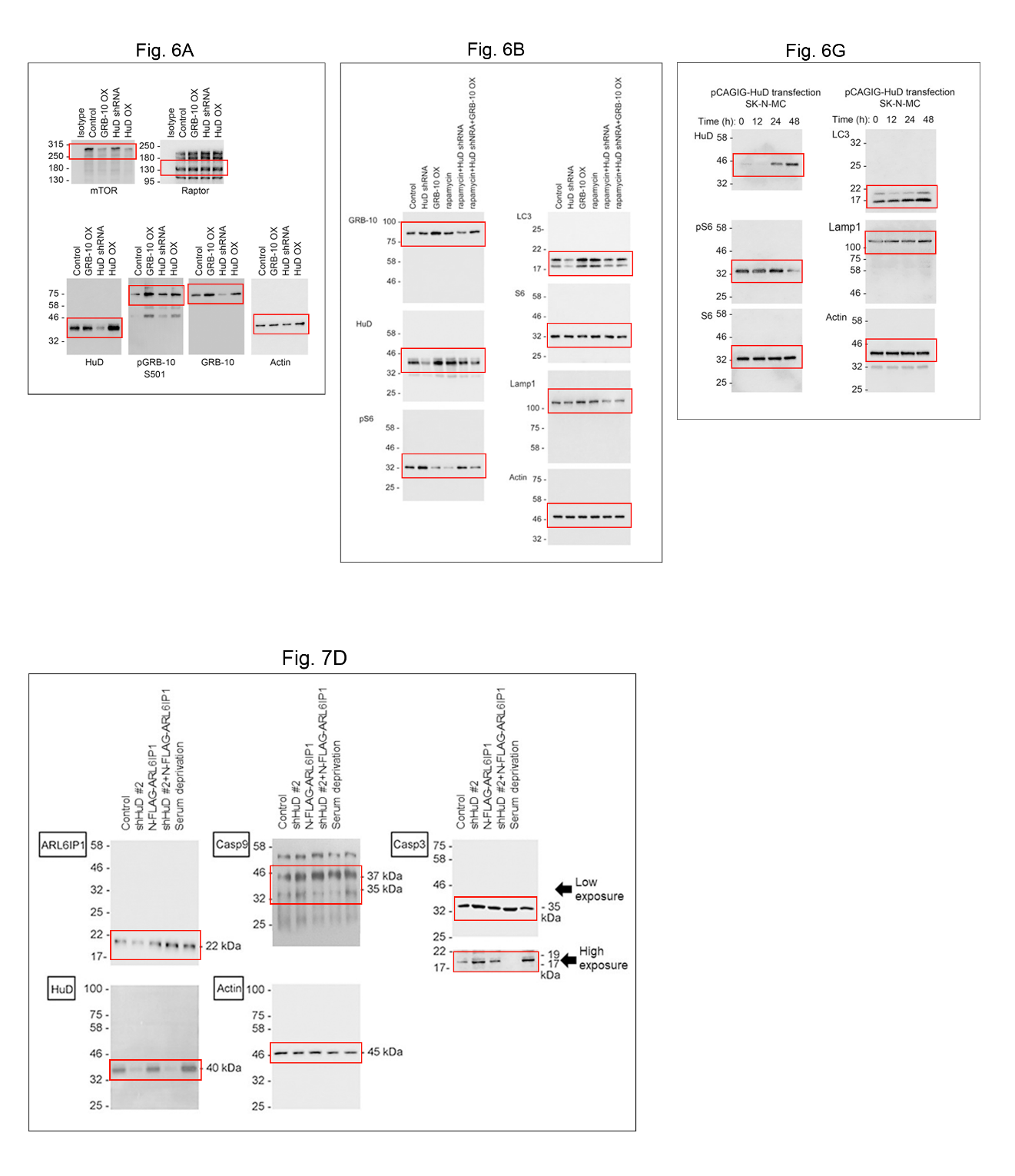


**Fig. S10** Raw Western blot images for Fig. 6 and 7

**Fig. S11**


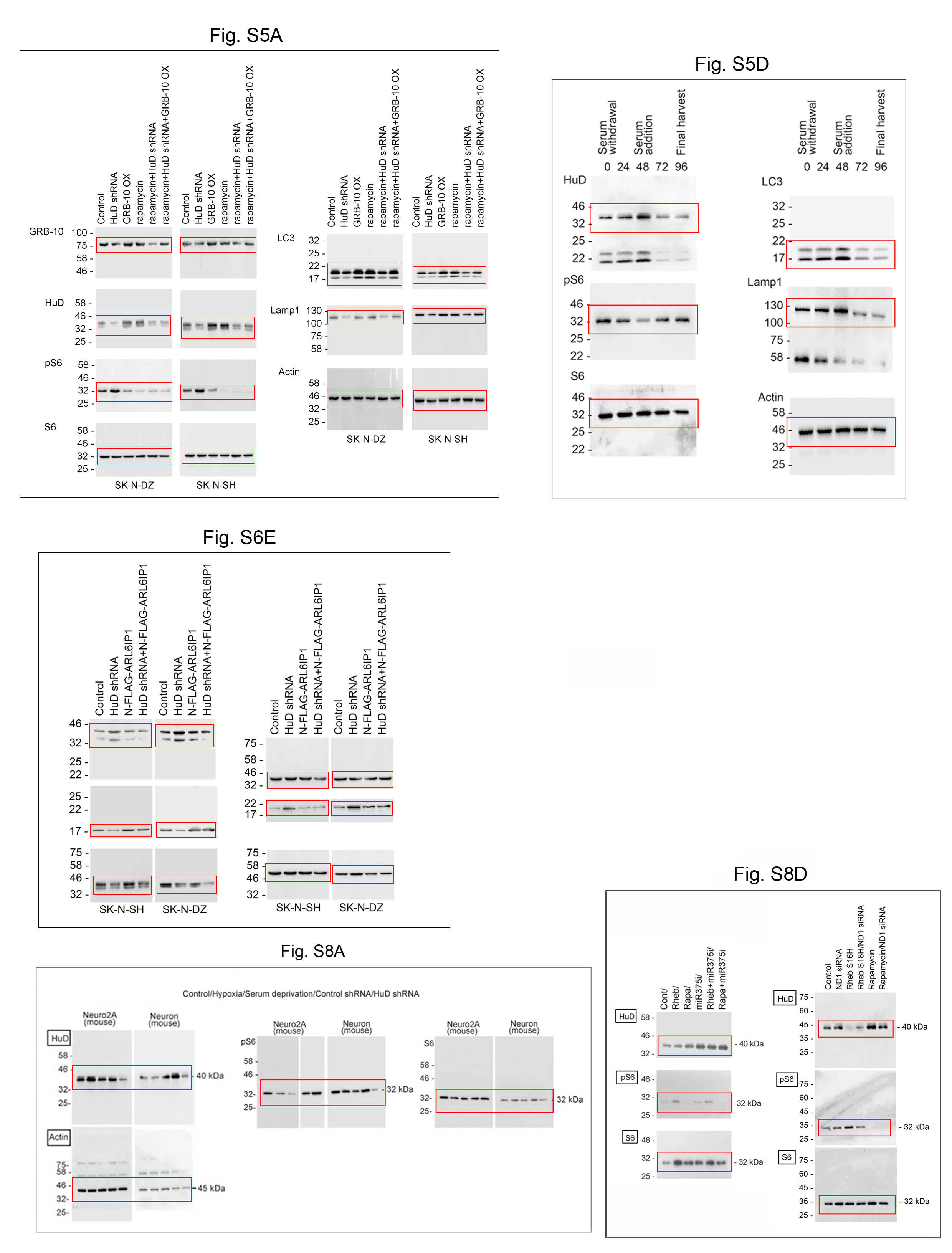


**Fig. S11** Raw Western blot images for Fig. S5, S6 and S8

**Supplemental methods**

**Cancer patient and cell line dataset analysis**

Expression of HuD, ACTB and GAPDH across different cancer types were curated from datasets available in R2 (R2: microarray analysis and visualization platform; http://r2.amc.nl; Department of Oncogenomics, Academic Medical Center) and by matching chip type and appropriate normalization schemes (hs, u133p2, MAS5.0). The data was plotted as mean±SD, significance for the population was calculated by ANOVA with “Tukey's Multiple Comparison Test”, where ***p<0.001. The detail of the datasets was listed in Table S4.

Neuroblastoma patient data were downloaded from R2 dataset and ranked according to HuD expression values. HuD bottom and top 10% patient populations were selected from the individual dataset and examined for expression of GRB-10 and ARL6IP1. Gene set comparison was executed within the individual dataset. Data were plotted as mean±SD. Significance for the population was performed by ANOVA with “Tukey's Multiple Comparison Test,” where ***p<0.001, **p<0.01, *p<0.05. The details about the dataset are described in Table S4.

HuD expression was compared for neuroblastoma and neural crest cells using the database “Mixed Neuroblastoma (MES-ADRN-Crest-Exp)-Versteeg-52” at R2. Data was plotted as mean±SD, with the significance for the population determined by ANOVA with “Tukey's Multiple Comparison Test,” where ***p<0.001.

Data for HuD expression in different cancer cell lines were obtained from the CCLE dataset (“The Cancer Cell Line Encyclopedia” by Broad Institute and the Novartis Institutes for Biomedical Research and its Genomics Institute of the Novartis Research Foundation, https://portals.broadinstitute.org/ccle).

**Cell culture**

Cells were grown at 37°C in a humidified 5% CO_2_ incubator. They were cultured in their respective media supplemented with 10% FBS, 50 U/ml penicillin, and 50 µg/ml streptomycin. The cell lines used were periodically checked for mycoplasma infection using Universal Mycoplasma Detection Kit (ATCC 30-1012K) (ATCC) and we found no cells that were infected. List of the cell lines listed in Table S5.

**Inducible HuD shRNA expressing stable cell line preparation**

The SMARTvector inducible HuD shRNA lenti-particles were purchased from Horizon-Dharmacon (CO, USA), The inducible vector contained TurboGFP (tGFP) reporter for visual tracking of expression upon doxycycline induction. The vector also expressed the puromycin resistance gene for antibiotic selection of transduced cells. The PuroR and Tet-On-3G (doxy regulated transactivator protein) were driven by the CMV promoter and tGFP and shRNA were driven by PTRE3G (inducible promoter with tetracycline response element, activated by Tet-On-3G). The IMR-32 and SK-N-SH cells were transduced with doxy-inducible HuD shRNA lentivirus particles (MOI 2.0) in presence of polybrene (5 μg/ml) in culture media. The transduced cells were selected with puromycin after 72 h post-transduction (selection dose for IMR-32 was 0.8 μg/ml and for SK-N-SH was 1.0 μg/ml) for 7 days. The selected cells were treated with doxycycline (2 μg/ml doxy) for 48 h to induce HuD shRNA and were checked for GFP fluorescence, HuD expression, and viability.

**Transfection of shRNA, siRNA, microRNAs, overexpression, and control vectors**

Transfection was performed according to the manufacturer’s protocol. Briefly, the cells were seeded in 6-well or 96-well cell culture plates in a growth medium without antibiotics. Lipofectamine-3000 (Thermo Fisher) was diluted in either DMEM or RPMI medium and mixed with the siRNA oligo or the vector construct and P3000 reagent (Thermo Fisher). The transfection complex was then mixed with media containing the cells. After 6 h, the cells were supplemented with fresh complete media and incubated for various times according to the assay. List of the plasmids were listed in Table S6.

**Lentivirus transduction**

Cells (3×10^4^) were seeded in a 96-well plate and HuD shRNA lentivirus particles were transduced at a concentration of MOI 2.0 in presence of polybrene (5 μg/ml) in the culture media. After incubation for 48 h, cells were assayed for viability. List of the lentiviral particle were listed in Table S7.

**Mouse cortical neuron isolation and culture**

Six-week-old mice were purchased from DBL Korea (South Korea). The mice were sacrificed with their cortical neurons isolated following a conventional anesthesia protocol. Briefly, the mouse brain was dissected and moved to HA media (Hibernate A media, Gibco A1247501). Cerebral meningeal was then removed and the cortex region was moved to HABG media (HA media+B27 media supplement). The tissue was then transferred to a 50 ml tube containing 5 ml HABG media and kept in a 30°C water bath for 8 min with shaking. Later, the tissue was transferred to 15 ml HABG media supplemented with 120 units papain and incubated for 30 min in a 37°C water bath with occasional shaking. Afterward, the tissue section was transferred to a 15 ml tube containing 2 ml HABG media and dissociated by repeated pipetting using a Pasteur pipette and the supernatant was collected and transferred to an OptiPrep density gradient (OptiPrep density 1.32; Sigma D1556). The mixture was then centrifuged at 1500g for 15 min at 22°C in a swing-bucket centrifuge. Gradient “fraction 3” from the top containing the neurons was collected and mixed with 5 ml HABG. The mixture was then centrifuged for 2 min at 200g and the pellet was collected; this step was repeated 2 times with washing. The cell pellet was resuspended in Neurobasal A media (Invitrogen 10888), supplemented with B27 (2%) and penicillin/streptomycin. These experiments involving animals were approved by the Institutional Animal Care and Use Committee (IACUC) of Hallym University, Chuncheon, South Korea (approval number, Hallym2020-19).

**Viability assay**

Cells were plated in a 96-well plate at a density of 3×10^4^ cells/well and after treatment, the assay was performed using ATCC ReliaBlue Cell Viability Reagent (ATCC 30-1014). Fluorescence (RFUs/absorbance at 570 nm) was measured and viability histograms were plotted using GraphPad Prism 5 software (GraphPad). All the experiments were conducted 2 or more times for reproducibility.

**RNA extraction and qPCR**

Total mRNA was extracted from various cell lines using the miRNeasy Mini Kit (Qiagen), from which, cDNA was made using the miScript II RT Kit (Qiagen) and according to the manufacturer’s protocol. Levels of the target message RNA were detected and quantified with SYBR Green RT-PCR kit (Qiagen). The PCR conditions were as follows: initial denaturation at 95°C for 15 min, 35 to 40 cycles of denaturation at 94°C for 30 sec, annealing at 55°C for 1 min and extension 72°C for 30 sec. The PCR was performed using Applied Biosystems StepOnePlus Real-Time PCR Systems (Applied Biosystems 4376599) with the built-in StepOnePlus software used to calculate fold changes to the control primers (2^ΔΔCT method). All the experiments were conducted 2 or more times for reproducibility. List of PCR primers were listed in Table S8.

**RNA immunoprecipitation assay and sequencing (RIP-SEQ)**

Cells were harvested and suspended in 1 ml crosslinking buffer (20 mM HEPES, 10 mM KCl, 1.5 mM MgCl_2_, and H_2_O) supplemented with 1% formaldehyde in phosphate-buffered saline (PBS). The mixture was incubated at room temperature for 10 min. Afterwards, 250 μl of 1 M glycine (final concentration, 0.25 M) was added and incubated for 5 min to quench the cross-linking reaction. Cells were washed with iced cold PBS three times and suspended in polysome lysis buffer (100 mM KCl, 5 mM MgCl_2_, 10 mM HEPES, pH 7.0, 0.5% NP40, 1 mM DTT, 100 unit/ml RNase Out, 400 μM VRC (vanadyl ribonucleoside complexes; Sigma-Aldrich 94742), 1X Protease inhibitor cocktail (Cell Signaling technology 5872), RNase inhibitors (50 units, Invitrogen RNaseOUT Recombinant Ribonuclease Inhibitor 10777019)) and incubated on ice for 5 min. The mRNP lysate was centrifuged at 15,000g for 15 min at 4°C to clear the lysate of large particles. The cleared supernatant was then transferred to a fresh microfuge tube, followed by pre-clearing of lysate with beads (Protein A/G PLUS-Agarose, Santa Cruz Biotechnology sc-2003) to reduce the background signals. The mRNP complex was then incubated with antibody-coated beads (IgG and HuD or FLAG) (Protein A/G PLUS-Agarose plus 1-2 µg antibody) in presence of 200 units of an RNase inhibitor (5 μl RNase Out), 2 μl vanadyl ribonucleoside complexes (to a final concentration of 400 μM), 10 μl of 100 mM DTT and 20 mM EDTA. The mixture was incubated for 6 h at 4°C with tumbling end over end. After the antibody binding, beads were washed 5 times with 1 ml of ice-cold NT2 buffer (50 mM Tris-HCl, pH 7.4, 150 mM NaCl, 1 mM MgCl_2_, 0.05% NP40). The beads were resuspended in 100 μl of NT2 buffer supplemented with 30 μg of proteinase K and incubated for 30 min at 55°C to release the RNP components. The released RNA was isolated and purified using the miRNeasy Mini Kit (Qiagen). The extracted RNAs were then either sent for sequencing to eBiogen, Seoul, South Korea, or cDNA was prepared using the miScript II RT Kit (Qiagen) and according to the manufacturer’s protocol. Levels of the target message RNA were detected and quantified with the SYBR Green RT-PCR kit (Qiagen) (1,2).

**RNA sequencing**

IMR-32 cells were treated with DMSO or rapamycin (25 nM) in addition to HuD shRNA for 24 h. Total RNA was extracted and quantified using a NANODROP Lite spectrophotometer (Thermo Fisher ND-ONE-W). Extracted RNA was sent to eBiogen Microarray Service (eBiogen, Seoul, South Korea) for Affymetrix Human Gene 2.0 ST Array profiling (Affymetrix, Thermo Fisher). Data were collected and normalized using Affymetrix Power Tools (APT) (Affymetrix, Thermo Fisher) and the Robust Multi-array Average (RMA) method [45,46]. Differentially expressed gene (DEG) analysis was also performed by eBiogen. Expression data were calculated as fold changes to the control.

**Protein extraction and Western blotting**

Cells were seeded in 6-well plates at 1×10^6^ cells per well. The treated cells were lysed on ice with radioimmunoprecipitation assay (RIPA) lysis buffer (150 mM NaCl, 1% NP-40, 0.5% sodium deoxycholate, 0.1% SDS, 50 mM Tris-HCl, pH 8) that included a cocktail of protease inhibitors (Roche, Basel, Switzerland). The cell lysates were then clarified by centrifugation at 4°C for 20 min at 13000g with the supernatants collected. The protein concentration of the lysates was measured by the Bradford assay (Bio-Rad). Equal amounts of protein were separated by sodium dodecyl sulfate-polyacrylamide gel electrophoresis (SDS-PAGE) (8-15% reducing gels) and then transferred onto polyvinylidene difluoride membranes (Millipore). After blocking the membrane with 5% non-fat milk (Difco/Becton Dickinson) in TBS (150 mM NaCl, 50 mM Tris-HCl, pH 7.6), it was incubated with the primary antibody overnight at 4°C and then washed with TBST (TBS, 0.1% Tween-20). The membrane was then incubated with the appropriate secondary antibody at room temperature for 3 h and washed again with TBST. The Western blots bands were visualized by enhanced chemiluminescence (ECL) (Luminata Forte) (Millipore) either by film or by detection FUSION FX-Western Blot & Chemi imaging system (Vilber Lourmat). The densitometry for the Western blot bands was performed using ImageJ software (NIH, nih.gov). All the experiments were conducted 2 or more times for reproducibility. Antibodies used for Western blotting and IP were listed in Table S9.

**Immunoprecipitation**

Cells were lysed on ice for 30 min in a Nonidet P40 (NP40) buffer (50 mM Tris-HCl, pH 7.4, 250 mM NaCl, 5 mM NaF, 1 mM Na_3_VO_4_, 1% NP40, 0.02% NaN_3_) containing a protease inhibitor cocktail (Roche). The cell lysates were then centrifuged at 13000g for 15 min at 4°C and the supernatants were collected. To preclear, the lysates, 1.5 mg of cell lysates were incubated with 100 μl of protein A/G PLUS-agarose (Santa Cruz Biotechnology sc-2003) for 3 h at 4°C. Lysates were then further incubated with anti-primary antibody (anti-GRB-10, Raptor, FLAG, ARL6IP1) overnight at 4°C with 20 μl protein A-agarose in 2 µg of antibodies. As a negative control, equivalent amounts of cellular lysates were incubated overnight without antibodies followed by bead incubation. The incubated beads were collected by centrifugation at 200g for 2 min at 4°C and were washed three times with the NP40 buffer. Samples were eluted from the beads by the addition of 50 μl 1X SDS sample buffer, immediately boiled, and were separated on 12% SDS-PAGE gels for Western blot analysis.

**RNA half-life assay**

IMR-32 shHuD inducible cells were plated and shHuD was induced by doxy (2 μg/ml) treatment or HEK293 cells transfected with shControl plasmid or shHuD and/or FLAG-HuD OX plasmids for 24 h. The cells were incubated and later treated with freshly prepared actinomycin D (5 μg/ml) added to the culture to stop the transcription process. Cells were collected at different time points (0, 1, 2, and 4 h). RNA was extracted using RNAzol reagent (Qiagen). Total mRNA was reverse transcribed into cDNA using an RNA reverse-transcription kit (Qiagen). RT-PCR was then performed as described in the “RNA extraction and qPCR” section; GRB-10 and ARL6IP1 mRNA levels were determined by qPCR and normalized by GAPDH; qPCR for HDAC2 reads were used as a negative control. Expression of GRB-10 and ARL6IP1 mRNA at the starting point was assigned a value of 1.0. The t1/2 was calculated from RNA level trend plots using GraphPad Prism 5 software. All the experiments were conducted 2 or more times for reproducibility.

**GST HuD pull-down assay for RNA stability**

IMR-32 cells were lysed in polysome lysis buffer (100 mM KCl, 5 mM MgCl2, 10 mM HEPES, pH 7, 0.5% NP40, 1 mM DTT, 100 unit/ml RNase Out, 400 μM VRC, vanadyl ribonucleoside complexes, Sigma-Aldrich 94742, 1X Protease inhibitor cocktail, Cell Signaling technology 5872, RNase inhibitors, 50 units, Invitrogen RNaseOUT™ Recombinant Ribonuclease Inhibitor 10777019) and incubated on ice for 5 min. The lysates were cleared by centrifugation at 14000g for 10 min at 4°C. The collected lysates were then precleared with 100 μl of glutathione−agarose (GA) beads (Millipore G4510) preloaded with 10 µg GST for 2 h at 4°C. Next, an RNA precipitation was performed with 20 μl of GA beads preloaded with 2 µg GST (Entrez Gene ID: 2944; NovusBio NBC1-18537) or 4 µg GST-HuD (giving equivalent molar amount as the control GST) (Entrez Gene ID: 1996; NovusBio H00001996-Q01). Afterwards, the beads were washed five times with ice-cold NT2 buffer (50 mM Tris-HCl, pH 7.4, 150 mM NaCl, 1 mM MgCl_2_, 0.05% NP40). The pull-down proteins were then digested with 30 μg of proteinase K and the released RNA was isolated and purified using the miRNeasy Mini Kit (Qiagen). Expression of HuD binders-GRB-10 and ARL6IP1 and nonbinder control, HDAC2, was performed in control and test samples as in the “RNA extraction and qPCR” section. The data was normalized by native HuD signal in the cells. The PCR conditions were as follows: initial denaturation at 95°C for 15 mins, followed by 35 to 40 cycles of denaturation at 94°C for 30 sec, annealing at 55°C for 1 min and extension 72°C for 30 sec. The PCR was performed using Applied Biosystems StepOnePlus™ Real-Time PCR Systems (Applied Biosystems 4376599); the built-in StepOnePlus software was used to calculate the fold changes with respect to reads from the control primers (2^ΔΔCT method). All the experiments were conducted 2 or more times for reproducibility.

**Ago2 binding assay**

Immunoprecipitations (IP) of Ago2-bound RNA-protein complexes were performed as previously described as in the RIP-SEQ assay section. Briefly, IMR-32 HuD shRNA cells were induced with 2 μg/ml doxy for 24 h and lysed in polysome lysis buffer (100 mM KCl, 5 mM MgCl_2_, 10 mM HEPES, pH 7, 0.5% NP40, 1 mM DTT, 100 unit/ml RNase Out, 400 μM VRC, vanadyl ribonucleoside complexes, Sigma-Aldrich 94742, 1X Protease inhibitor cocktail, Cell Signaling technology 5872, RNase inhibitors of 50 units, Invitrogen RNaseOUT™ Recombinant Ribonuclease Inhibitor 10777019). The cleared supernatants were incubated with 2 µg of anti-Ago2 or IgG primary antibody and 20 µl A/G PLUS-agarose beads at 4°C for 6 h. The details of the Ago2 antibody are available in Table S9. After washing with NT2 buffer (50 mM Tris-HCl, pH 7.4, 150 mM NaCl, 1 mM MgCl_2_, 0.05% NP40), the bead-bound Ago2-RNA or IgG-RNA complexes were assayed for bound RNA by qPCR. All the experiments were conducted 2 or more times for reproducibility.

**Biotinylated RNA pull down assay**

RNA-protein interaction was detected by RNA pulldown and immunoblotting method. 5’ end biotin labeled RNA oligoes were obtained from Bioneer Corporation. RNA oligoes at 100 pmol were immobilized on 5 μl streptavidin agarose beads. IMR-32 and SK-N-SH cells were washed with pre chilled PBS twice and gently resuspended in hypotonic buffer solution (20 mM Tris-HCl, pH 7.4, 10 mM NaCl, 3 mM MgCl_2_). Cells were lysed with NP40 (10%) by rigorous votexing. The mixture was then centrifuged at 3000 rpm at 4 °C and the nuclear extract was collected as pellet and resuspended in NP40 and quantified. RNA-bead conjugates were then incubated in nuclear extracts of IMR-32 or SK-N-SH cells at 4 °C overnight under 100 RPM rotation in a rotator. The beads were then washed 3 times and heated at 80 °C for 5 min in 1X protein loading dye. Levels of bound HuD were then detected by Western blotting.

**Immunocytochemistry (ICC)**

Cells were plated at 1×10^5^ cells per well, grown on round coverslips in 24-well dishes. After treatment with different reagents, the cells were washed with ice-cold PBS. They were next fixed with 4% paraformaldehyde (PFA) solution for 15 min at room temperature and washed twice with PBS. The cells were next permeabilized with PBS containing 0.25% Triton X-100 for 10 min and washed three times for 5 min each with PBS and incubated in 10% goat serum in PBS for 30 min to block nonspecific antibody binding. The cells were then incubated overnight at 4°C with the diluted antibody in 3% BSA in PBS. The cells were then washed three times for 5 min with PBS and incubated with the secondary antibody (1:1000) with DAPI (0.1 μg/ml) in 1% BSA for 1 h at room temperature in dark. After washing three times for 5 min each in PBS, a coverslip was mounted onto a slide with DPX Mounting Medium (Dako North America) and dried at room temperature. The images of the cells were captured using a confocal microscope (Carl Zeiss LSM 710) and analyzed with ZEN 2.6 software (Carl Zeiss). The antibodies used for ICC were listed in Table S10.

**Immunohistochemistry (IHC-F)**

Tumor tissues were fixed in 4% PFA in PBS overnight at 4°C. The fixed tissues were sequentially dipped in 15% and 30% sucrose solution in PBS, each overnight at 4°C. The tissues were mounted in OCT blocks and frozen for 48 h at -80°C. The OCT blocks were sectioned at 5 μm thickness using a cryo-sectioning machine. The cold slides were kept at room temperature for 10 min before starting the experiment; antigen retrieval was performed with 0.05% trypsin in PBS for 15 min at 37°C. After washing and permeabilization, slides were blocked in 10% goat serum in PBS. Incubation with primary antibodies and isotype controls was performed at 4°C overnight. The slides were then incubated in Alexa Fluor-conjugated secondary antibodies with DAPI for 2 h at room temperature. After washing three times for 5 min each in PBS, a coverslip was mounted onto a slide with DPX Mounting Medium (Dako North America). The images of the cells were captured using a confocal microscope (Carl Zeiss LSM 710) and analyzed in ZEN 2.6 software (Carl Zeiss). The antibodies used for IHC-F were listed in Table S11.

**Neuroblastoma tissue array staining**

Paraffin-embedded neuroblastoma and peripheral nerve tissue array were commercially obtained (Biomax NB642a). Slides were heated in an oven at 56°C for 15 min; they were then transferred to a xylene bath for 5 min and two changes were performed. The slides were then rehydrated further using gradient alcohol changes. Antigen retrieval was performed with 0.05% trypsin in PBS for 15 min at 37°C water bath. After washing and permeabilization, the slides were blocked in 10% goat serum in PBS. Incubation with primary antibodies and isotype controls was performed at 4°C overnight. The slides were stained with anti-HuD and pS6K antibodies listed in the “Immunocytochemistry (IHC-F)” section. The slides were then incubated with Alexa Fluor-conjugated secondary antibodies for 2 h at room temperature, followed by DAPI staining. After washing three times for 5 min each in PBS, a coverslip was mounted onto a slide with DPX Mounting Medium (Dako North America). Images of the slides were obtained using a confocal microscope (Carl Zeiss LSM 710) and analyzed with ZEN 2.6 software (Carl Zeiss).

**Xenograft study in nude mice**

Doxy-inducible HuD shRNA expressing IMR-32 and SK-N-SH cells were cultured, harvested, and suspended in 50% matrigel (Trevigen 3433-001-R1) in PBS. Each with 1×10^7^ cells were inoculated subcutaneously in the right flank region of 5-week old nude, athymic mice (nu/nu) obtained from DBL Korea (South Korea). Treatment started at day 10 post tumor implantation. The mice were injected intraperitoneally with doxy at 2 mg/kg body weight on alternate days, three times a week, in endotoxin-free 100 μl of PBS for 30 days. The control group received PBS. Tumors were assessed using a digital caliper and tumor volume was calculated according to the formula (length ×width^2/ 2). The tumors were isolated and treated with RNAzol (Qiagen) for RNA extraction, with RIPA buffer for protein extraction and with 4% PFA in PBS for immunohistochemistry. The mice were kept under pathogen-free conditions in housing with ambient 12-h light/day cycle, 22±2°C room temperature and 50±10% humidity. Food and water were made available at all times; the mice feeds were procured from a commercial food company (Purina, South Korea). The experiments involving these animals were approved by the Institutional Animal Care and Use Committee (IACUC) of Hallym University, Chuncheon, South Korea (approval number, Hallym2019-51).

**Statistical analysis**

Statistical analyses were performed using GraphPad Prism 5 software (GraphPad). The analytical data are presented as mean±SEM and compared statistically by the tests indicated. For all, p<0.05 was considered as being statistically significant.

**Supplementary tables**

**Table S1:** List of cancer patient datasets

| No. | Cancer type | ID | Number of patients |
| --- | --- | --- | --- |
| 1 | Neuroblastoma | GEO- GSE45547 | 649 |
| 2 | Neuroblastoma | GEO- GSE49710 | 498 |
| 3 | Neuroblastoma | TARGET-NB | 249 |
| 4 | Neuroblastoma | GEO-GSE16476 | 88 |
| 5 | Lung (NSCLC) | GEO-GSE63074 | 410 |
| 6 | Ewing’s sarcoma | GEO-GSE34620 | 117 |
| 7 | Breast cancer | GEO- GSE3494 | 251 |
| 8 | Kidney cancer | GEO- GSE2109 | 261 |
| 9 | Multiple Myeloma | GEO- GSE2658 | 542 |
| 10 | Prostate cancer | GEO- GSE6956 | 89 |
| 11 | Glioblastoma | TCGA-540 | 540 |
| 12 | Leukemia | GEO- GSE13159 | 2,004 |

**Table S2:** List of cell lines

| No. | Cell name | Vendor | Cell type | Culture media |
| --- | --- | --- | --- | --- |
| 1 | IMR-32 | KCLB, South Korea | Neuroblastoma (Hu) | RPMI; 10% FBS |
| 2 | SK-N-MC | KCLB, South Korea | Neuroblastoma (Hu) | DMEM; 10% FBS |
| 3 | SK-N-DZ | ATCC, USA | Neuroblastoma (Hu) | DMEM; 10% FBS |
| 4 | SK-N-SH | KCLB, South Korea | Neuroblastoma (Hu) | DMEM; 10% FBS |
| 5 | SH-SY5Y | KCLB, South Korea | Neuroblastoma (Hu) | DMEM; 10% FBS |
| 6 | Neuro2A | JCRB, Japan | Neuroblastoma (Mus) | DMEM; 10% FBS |
| 7 | NCI-H146 | KCLB, South Korea | Small cell lung cancer (Hu) | RPMI; 10% FBS |
| 8 | NCI-H69 | KCLB, South Korea | Small cell lung cancer (Hu) | RPMI; 10% FBS |
| 9 | NCI-H889 | KCLB, South Korea | Small cell lung cancer (Hu) | RPMI; 10% FBS |
| 10 | NCI-H209 | KCLB, South Korea | Small cell lung cancer (Hu) | RPMI; 10% FBS |
| 11 | HEK293 | ATCC, USA | Embryonic kidney with SV-40-T antigen (Hu) | DMEM; 10% FBS |

Hu, human cell line; Mus, mouse cell line

**Table S3:** List of plasmids

| No. | Expression vector | Insert | Remarks |
| --- | --- | --- | --- |
| 1 | pcDNA3-FLAG vector | GRB10 (Hu) (Entrez Gene ID: 2887) | Addgene plasmid # 37481 |
| 2 | pFRT-TODest-FLAGHA | HuB (Hu) (Entrez Gene ID: 1993) | Addgene plasmid # 65755 |
| 3 | pFRT-TODest-FLAGHA | HuC (Hu) (Entrez Gene ID: 1995) | Addgene plasmid # 65756 |
| 4 | pCMV3-C-Myc | HuD (Hu) (Entrez Gene ID: 1996) | C-terminal Myc tag HuD vector |
| 5 | pCMV3-N-FLAG | HuD (Hu) (Entrez Gene ID: 1996) | N-terminal FLAG tag HuD vector |
| 6 | pCMV3-N-FLAG | Control FLAG vector | N-terminal FLAG tag control vector |
| 7 | pCMV3-C-MYC | Control Myc vector | C-terminal Myc tag control vector |
| 8 | pCAGIG (GFP) | HuD (Hu) (Entrez Gene ID: 1996) | Bicistronic GFP and HuD vector |
| 9 | pCAGIG (GFP) | GFP control vector | Bicistronic GFP control vector |
| 10 | pCAGIG (GFP) | NEUROD1 (Hu) (Entrez Gene ID: 4760) | Bicistronic GFP and NeuroD1 vector |
| 11 | pCMV3-MYC | HuD (Hu) MT | shRNA-binding-region silent mutation |
| 12 | pCMV3-FLAG | HuD (Hu) MT | shRNA-binding-region silent mutation |
| 13 | pmCherry (RFP) | HuD (Mus) (Entrez Gene ID: 15572) | Bicistronic RFP and HuD vector |
| 14 | pCMV3-N-FLAG | HuD (Hu) MT | N-terminal FLAG tag HuD vector with RBD 1 mutation |
| 15 | pCMV3-N-FLAG | HuD (Hu) MT | N-terminal FLAG tag HuD vector with RBD 2 mutation |
| 16 | pCMV3-N-FLAG | HuD (Hu) MT | N-terminal FLAG tag HuD vector with RBD 3 mutation |
| 17 | pCMV3-N-FLAG | HuD (Hu) MT | N-terminal FLAG tag HuD vector with RBD 1 and 2 mutation |
| 18 | pCMV3-N-FLAG | HuD (Hu) MT | N-terminal FLAG tag HuD vector with RBD 1, 2 and 3 mutation |
| 19 | pGPH1/GFP/Neo | HuD shRNA#1 | Bicistronic GFP containing HuD silencing vector 1, expressing ATCAGGGATGCTAACCTCTAT |
| 20 | pGPH1/GFP/Neo | HuD shRNA#2 | Bicistronic GFP containing HuD silencing vector 2, expressing GAATGAACATCCCTGGTCACA |
| 21 | pGPH1/GFP/Neo | Control | Bicistronic GFP control shRNA vector |
| 22 | pCMV3-N-FLAG | GRB10 (Hu) (Entrez Gene ID: 2887) | N-terminal FLAG tag GRB10 vector |
| 23 | miR375 mimic | miR375 | Qiagen MSY0000728 |
| 24 | miR375 inhibitor | miR375 anti-oligo | Qiagen MIN0000728 |
| 25 | GRB10 siRNA | GRB10 siRNA | Santa Cruz Biotechnology |
| 26 | pCMV3-N-FLAG | ARL6IP1 (Hu) (Entrez Gene ID: 23204) | N-terminal FLAG tag ARL6IP1 vector |
| 27 | ARL6IP1 siRNA | ARL6IP1 siRNA | Santa Cruz Biotechnology |

**Table S4:** List of lentiviral particles

| No. | Expression vector | Insert | Remarks |
| --- | --- | --- | --- |
| 1 | Control shRNA Lentiviral Particles-A | Control shRNA | Santa Cruz Biotechnology (sc-108080) |
| 2 | HuD shRNA (h) Lentiviral Particles | HuD shRNA | Santa Cruz Biotechnology (sc-37835-V) |
| 3 | Doxy inducible HuD shRNA (h) Lentiviral Particles  HuD shRNA #2 | HuD shRNA#2 | Doxy inducible vector custom made by Dharmacon; expressing GAATGAACATCCCTGGTCACA |

**Table S5:** List of primers for qPCR

| No. | Gene | Vendor | Cat. No./ primer sequence |
| --- | --- | --- | --- |
| 1 | Human HuD | Qiagen | PPH09946A |
| 2 | Human HuR | Qiagen | PPH13883B |
| 3 | Human HuC | Qiagen | PPH07095A |
| 4 | Human HuB | Qiagen | LPH31286A |
| 5 | Human GRB-10 | Qiagen | PPH05866B |
| 6 | Human GAP43 | Qiagen | QT00023639 |
| 7 | Human CamKIIa | Qiagen | QT00024010 |
| 8 | Human NeuroD1 | Qiagen | PPH00039E |
| 9 | Human miR375 | Qiagen | MS00031829 |
| 10 | Human GAPDH | Qiagen | QT00079247/ PPM02946E |
| 11 | Human GRB10 | Bioneer | F-5’ AACTAAAGAGCTGAGGTTGC  R-5’ TTCTGGTAAAGGAGCATTCC |
| 12 | Human HuD | Bioneer | F-5’ ATCCTGCAAACTTGTGAGAG  R-5’ AAAGTGTTGATGGCTTTCTC |
| 13 | Human GAPDH | Bioneer | F-5’ GTCTCCTCTGACTTCAACAG  R-5’ CTGTAGCCAAATTCGTTGTC |
| 14 | Human ARL6IP1 | Bioneer | F-5’ CGCCTCTTCACACTAAAGG  R-5’AGGTTGTGGACTTGTTGTC |
| 15 | Human BGLAP | Bioneer | F-5’ ACACTCCTCGCCCTATTG  R-5’ CTCCTGCTTGGACACAAAG |
| 16 | Human CDCA3 | Bioneer | F-5’ GTACCCAGTTATCTGTTGAGG  R-5’ GTTTCTGTGGGCTGTCTTG |
| 17 | Human CENPN | Bioneer | F-5’ GACTGTAAATTTCCGACAGAG  R-5’ AATGATGTCTAACAGGGCAG |
| 18 | Human DEPDC1B | Bioneer | F-5’ GCAGGGAGTTGTTATTCTTG  R-5’ CCAAGTACATAGGCTGCTTC |
| 19 | Human MAD2L1 | Bioneer | F-5’ GGCAGTTTGATATTGAGTGTG  R-5’ CGTAGCTGTGATCTGTCTG |
| 20 | Human UBE2T | Bioneer | F-5’ TGTGTTGACCTCTATTCAGC  R-5’ ACTGTCTGGCATTCTTGAG |

**Table S6:** List of antibodies for Western blotting and IP

| No. | Antibody | Applications | Vendor | Cat. No. |
| --- | --- | --- | --- | --- |
| 1 | HuD | WB, ICC, IHC-F | Santa Cruz Biotechnology | sc-28299 |
| 2 | S6 | WB | Cell Signaling Technology | 2217 |
| 3 | pS6 | WB | Cell Signaling Technology | 5364 |
| 4 | EGF receptor (Endogenous) | WB | Cell Signaling Technology | 4267S |
| 5 | p-EGF receptor (Y1068) | WB | Cell Signaling Technology | 2234S |
| 6 | GRB10 | WB, IP | Cell Signaling Technology | 3702S |
| 7 | LC3A/B (D3U4C) XP® | WB, ICC | Cell Signaling Technology | 12741 |
| 8 | Raptor | WB, IP | Abcam | ab5454 |
| 9 | Lamp1 | WB, ICC | Abcam | ab25245 |
| 10 | β-actin | WB | Cell Signaling Technology | 4967 |
| 11 | Ago2 | WB, IP | Abcam | ab186733 |
| 12 | FLAG | WB, IP | Sigma | F7425 |
| 13 | Akt | WB | Cell Signaling Technology | 9272 |
| 14 | pAkt | WB | Cell Signaling Technology | 4060 |
| 15 | ARL6IP1 | WB, ICC | Santa Cruz Biotechnology | sc-514227 |
| 16 | Caspase 9 | WB | Cell Signaling Technology | 9502 |
| 17 | Caspase 3 | WB | Cell Signaling Technology | 14220 |

WB-Western blot; ICC-immunocytochemistry; IHC-F-immunohistochemistry-fluorescence; IP- immunoprecipitation.

**Table S7:** List of antibodies for ICC

| No. | Antibody | Applications | Vendor | Cat. No. |
| --- | --- | --- | --- | --- |
| 1 | HuD | WB, ICC, IHC-F | Santa Cruz Biotechnology | sc-28299 |
| 2 | GRB10 | ICC | Atlas antibodies | HPA031818 |
| 3 | LC3A/B (D3U4C) XP | WB, ICC | Cell Signaling Technology | 12741 |
| 4 | Calreticulin-Alexa Fluor 647 | ICC | Abcam | ab196159 |
| 5 | ARL6IP1 | WB, ICC | Santa Cruz Biotechnology | sc-514227 |

WB-Western blot; ICC-immunocytochemistry; IHC-F-immunohistochemistry-fluorescence; IP- immunoprecipitation

**Table S8:** List of antibodies for IHC-F

| No. | Antibody | Applications | Vendor | Cat. No. |
| --- | --- | --- | --- | --- |
| 1 | HuD | WB, ICC, IHC-F | Santa Cruz Biotechnology | sc-28299 |
| 2 | pS6K | IHC-F | Abclonal | AP0564 |
| 3 | Glypican-2- Alexa Fluor 647 | IHC-F | Santa Cruz Biotechnology | sc-53831 AF647 |
| 4 | Ki67 | IHC-F | Abcam | ab15580 |
| 5 | GFP | IHC-F | Abcam | ab13970 |

WB-Western blot; ICC-immunocytochemistry; IHC-F-immunohistochemistry-fluorescence; IP- immunoprecipitation

**Table S9:** Listing of Biomax neuroblastoma tissue array (Biomax, NB642a)

| Pos. | No. | Age | Sex | Organ/Anatomic Site | Pathology diagnosis | Stage | Type |
| --- | --- | --- | --- | --- | --- | --- | --- |
| A1 | 1 | 3 | F | Retroperitoneum | Neuroblastoma | I | Malignant |
| A2 | 2 | 3 | F | Retroperitoneum | Neuroblastoma | I | Malignant |
| A3 | 3 | 8 | F | Retroperitoneum | Neuroblastoma | I | Malignant |
| A4 | 4 | 8 | F | Retroperitoneum | Neuroblastoma | I | Malignant |
| A5 | 5 | 1 | F | Retroperitoneum | Neuroblastoma | I | Malignant |
| A6 | 6 | 1 | F | Retroperitoneum | Neuroblastoma | I | Malignant |
| A7 | 7 | 18 | M | Retroperitoneum | Neuroblastoma | I | Malignant |
| A8 | 8 | 18 | M | Retroperitoneum | Neuroblastoma | I | Malignant |
| A9 | 9 | 27 | M | Retroperitoneum | Neuroblastoma | IV | Malignant |
| A10 | 10 | 27 | M | Retroperitoneum | Neuroblastoma | IV | Malignant |
| B1 | 11 | 7 | M | Retroperitoneum | Neuroblastoma | IV | Malignant |
| B2 | 12 | 7 | M | Retroperitoneum | Neuroblastoma | IV | Malignant |
| B3 | 13 | 4 | F | Retroperitoneum | Neuroblastoma | IIB | Malignant |
| B4 | 14 | 4 | F | Retroperitoneum | Neuroblastoma | IIB | Malignant |
| B5 | 15 | 6 | M | Retroperitoneum | Neuroblastoma | IV | Malignant |
| B6 | 16 | 6 | M | Retroperitoneum | Neuroblastoma | IV | Malignant |
| B7 | 17 | 26 | F | Retroperitoneum | Neuroblastoma | I | Malignant |
| B8 | 18 | 26 | F | Retroperitoneum | Neuroblastoma | I | Malignant |
| B9 | 19 | 3 | F | Retroperitoneum | Neuroblastoma | I | Malignant |
| B10 | 20 | 3 | F | Retroperitoneum | Neuroblastoma | I | Malignant |
| C1 | 21 | 4 | F | Retroperitoneum | Neuroblastoma | IV | Malignant |
| C2 | 22 | 4 | F | Retroperitoneum | Neuroblastoma | IV | Malignant |
| C3 | 23 | 1 | F | Retroperitoneum | Neuroblastoma | I | Malignant |
| C4 | 24 | 1 | F | Retroperitoneum | Neuroblastoma | I | Malignant |
| C5 | 25 | 84 | M | Retroperitoneum | Neuroblastoma | II | Malignant |
| C6 | 26 | 84 | M | Retroperitoneum | Neuroblastoma | II | Malignant |
| C7 | 27 | 2 | F | Retroperitoneum | Neuroblastoma | I | Malignant |
| C8 | 28 | 2 | F | Retroperitoneum | Neuroblastoma | I | Malignant |
| C9 | 29 | 5 | M | Retroperitoneum | Neuroblastoma | I | Malignant |
| C10 | 30 | 5 | M | Retroperitoneum | Neuroblastoma | I | Malignant |
| D1 | 31 | 4 | F | Retroperitoneum | Neuroblastoma | I | Malignant |
| D2 | 32 | 4 | F | Retroperitoneum | Neuroblastoma | I | Malignant |
| D3 | 33 | 8 Mon. | M | Retroperitoneum | Neuroblastoma | IV | Malignant |
| D4 | 34 | 8 Mon. | M | Retroperitoneum | Neuroblastoma | IV | Malignant |
| D5 | 35 | 1 | M | Retroperitoneum | Neuroblastoma | IV | Malignant |
| D6 | 36 | 1 | M | Retroperitoneum | Neuroblastoma | IV | Malignant |
| D7 | 37 | 4 | M | Retroperitoneum | Neuroblastoma | III | Malignant |
| D8 | 38 | 4 | M | Retroperitoneum | Neuroblastoma | III | Malignant |
| D9 | 39 | 1 | F | Mediastinum | Neuroblastoma | I | Malignant |
| D10 | 40 | 1 | F | Mediastinum | Neuroblastoma | I | Malignant |
| E1 | 41 | 2 | F | Mediastinum | Neuroblastoma | I | Malignant |
| E2 | 42 | 2 | F | Mediastinum | Neuroblastoma | I | Malignant |
| E3 | 43 | 51 | M | Mediastinum | Neuroblastoma | I | Malignant |
| E4 | 44 | 51 | M | Mediastinum | Neuroblastoma | I | Malignant |
| E5 | 45 | 25 | F | Pelvic cavity | Neuroblastoma | I | Malignant |
| E6 | 46 | 25 | F | Pelvic cavity | Neuroblastoma | I | Malignant |
| E7 | 47 | 6 | M | Adrenal gland | Neuroblastoma | I | Malignant |
| E8 | 48 | 6 | M | Adrenal gland | Neuroblastoma | I | Malignant |
| E9 | 49 | 20 | M | Adrenal gland | Neuroblastoma | I | Malignant |
| E10 | 50 | 20 | M | Adrenal gland | Neuroblastoma | I | Malignant |
| F1 | 51 | 6 | M | Adrenal gland | Neuroblastoma | I | Malignant |
| F2 | 52 | 6 | M | Adrenal gland | Neuroblastoma | I | Malignant |
| F3 | 53 | 5 | F | Adrenal gland | Neuroblastoma | I | Malignant |
| F4 | 54 | 5 | F | Adrenal gland | Neuroblastoma | I | Malignant |
| F5 | 55 | 31 | M | Nerve | Peripheral nerve tissue | - | Normal |
| F6 | 56 | 31 | M | Nerve | Peripheral nerve tissue | - | Normal |
| F7 | 57 | 36 | M | Nerve | Peripheral nerve tissue | - | Normal |
| F8 | 58 | 36 | M | Nerve | Peripheral nerve tissue | - | Normal |
| F9 | 59 | 33 | M | Nerve | Peripheral nerve tissue | - | Normal |
| F10 | 60 | 33 | M | Nerve | Peripheral nerve tissue | - | Normal |
| G1 | 61 | 25 | M | Nerve | Peripheral nerve tissue | - | Normal |
| G2 | 62 | 25 | M | Nerve | Peripheral nerve tissue | - | Normal |
| G3 | 63 | 32 | M | Nerve | Peripheral nerve tissue | - | Normal |
| G4 | 64 | 32 | M | Nerve | Peripheral nerve tissue | - | Normal |

**Table S10:** Top enriched Gene Ontology terms for HuD mRNA targets by RIP-SEQ (with chemical crosslinking) in normal, cell growth condition

| GO TERMS | -LOG10 (p-value) |
| --- | --- |
| Transport Golgi to ER | 1.806368 |
| COPII vesicle coating | 1.811877 |
| Fatty acid biosynthetic | 1.882352 |
| Amino acid metabolic | 1.890551 |
| Positive germinal center formation | 1.925611 |
| Phosphorylation | 1.933328 |
| Response to DNA damage stimulus | 1.934311 |
| Endosomal transport | 2.039035 |
| Saturated fatty acid | 2.117313 |
| Monounsaturated fatty acid | 2.117313 |
| Polyunsaturated fatty acid | 2.117313 |
| DNA replication-independent nucleosome | 2.27754 |
| Megakaryocyte differentiation | 2.335798 |
| Protein secretion | 2.440069 |
| Protein transport | 2.526961 |

**Table S11:** Top enriched Gene Ontology terms for HuD mRNA targets by RIP-SEQ sequencing in serum starvation

| GO TERMS | -LOG10 (p-value) |
| --- | --- |
| Transcription initiation | 1.887722 |
| CENP-A nucleosome assembly | 1.974424 |
| Gene expression (Signal transduction) | 2.022741 |
| DNA replication-independent nucleosome | 2.043613 |
| DNA replication-dependent nucleosome | 2.173503 |
| Proteasomal-protein catabolic | 2.255028 |
| Cellular protein metabolic | 2.339837 |
| Regulation of cytochrome c mitochondria | 2.517921 |
| Gene expression (epigenetic) | 2.585154 |
| Protein heterotetramerization | 2.591213 |
| Chromatin silencing | 2.950878 |
| Telomere organization | 3.34481 |
| Regulation of gene expression | 3.409008 |
| Chromatin silencing at rDNA | 4.418728 |
| Nucleosome assembly | 4.947242 |

**Table S12:** Top enriched Gene Ontology terms for global cell transcript changes post HuD silencing are related to cell division, cell cycle, proliferation, and ER stress response

| GO TERMS | -LOG10 (p-value) |
| --- | --- |
| Mitotic chromosome condensation | 6.083842 |
| Cell proliferation | 6.487188 |
| Mitotic cytokinesis | 6.506967 |
| Response to ER stress | 6.626479 |
| Cholesterol biosynthetic | 6.627155 |
| Metaphase plate congression | 6.790626 |
| CENP-A nucleosome assembly | 7.220521 |
| Microtubule-based movement | 8.080693 |
| Mitotic sister chromatid segregation | 9.819006 |
| Chromosome segregation | 10.19063 |
| G2/M transition of mitotic cell cycle | 10.78657 |
| Nucleosome assembly | 12.98814 |
| Sister chromatid cohesion | 15.48039 |
| Mitotic nuclear division | 21.95129 |
| Cell division | 29.23123 |
